# Physical therapy interventions for older people with vertigo, dizziness and balance disorders addressing mobility and participation: a systematic review

**DOI:** 10.1186/s12877-020-01899-9

**Published:** 2020-11-23

**Authors:** Verena Regauer, Eva Seckler, Martin Müller, Petra Bauer

**Affiliations:** 1 Centre for Research, Development and Technology Transfer, Rosenheim Technical University of Applied Sciences, Hochschulstraße 1, 83024 Rosenheim, Germany; 2 Institute for Medical Information Processing, Biometry and Epidemiology, Ludwig Maximilian University of Munich, Marchioninistraße 17, 81377 Munich, Germany; 3 Faculty for Applied Health and Social Sciences and Centre for Research, Research, Development and Technology Transfer, Rosenheim Technical University of Applied Sciences, Hochschulstraße 1, 83024 Rosenheim, Germany

**Keywords:** Aged, Aged, 80 and over, Dizziness, Mobility limitation, Nervous system diseases, Physical therapy modalities, Postural balance, Social participation, Treatment outcome, Vertigo

## Abstract

**Background:**

Vertigo, dizziness and balance disorders (VDB) are among the most relevant contributors to the burden of disability among older adults living in the community and associated with immobility, limitations of activities of daily living and decreased participation. The aim of this study was to identify the quality of evidence of physical therapy interventions that address mobility and participation in older patients with VDB and to characterize the used primary and secondary outcomes.

**Methods:**

A systematic search via MEDLINE (PubMed), Cochrane Library, CINAHL, PEDro, forward citation tracing and hand search was conducted initially in 11/2017 and updated in 7/2019. We included individual and cluster-randomized controlled trials and trials with quasi-experimental design, published between 2007 and 2017/2019 and including individuals ≥65 years with VDB. Physical therapy and related interventions were reviewed with no restrictions to outcome measurement. Screening of titles, abstracts and full texts, data extraction and critical appraisal was conducted by two independent researchers. The included studies were heterogeneous in terms of interventions and outcome measures. Therefore, a narrative synthesis was conducted.

**Results:**

A total of 20 randomized and 2 non-randomized controlled trials with 1876 patients met the inclusion criteria. The included studies were heterogeneous in terms of complexity of interventions, outcome measures and methodological quality. Vestibular rehabilitation (VR) was examined in twelve studies, computer-assisted VR (CAVR) in five, Tai Chi as VR (TCVR) in three, canal repositioning manoeuvres (CRM) in one and manual therapy (MT) in one study. Mixed effects were found regarding body structure/function and activities/participation. Quality of life and/or falls were assessed, with no differences between groups. VR is with moderate quality of evidence superior to usual care to improve balance, mobility and symptoms.

**Conclusion:**

To treat older individuals with VDB, VR in any variation and in addition to CRMs seems to be effective. High-quality randomized trials need to be conducted to inform clinical decision making.

**Trial registration:**

PROSPERO 2017 CRD42017080291.

**Supplementary Information:**

The online version contains supplementary material available at 10.1186/s12877-020-01899-9.

## Background

Vertigo, dizziness and balance disorders (VDB) are the most relevant factors influencing the burden of disability among older adults [1] and are associated with immobility, limitations in activities of daily living (ADL) and decreased participation [2, 3]. VDB are frequent complaints of older people [4–7] with a reported prevalence up to 50% [8, 9], and the prevalence tends to increase with age [10]. Complaints of VDB are distinct risk factors for falls [11], and even the fear of falling may lead to activity restriction and disability [12]. Especially in older individuals, a unique underlying cause of VDB is difficult to determine because of multifactorial potential underlying pathomechanisms in the vestibular, visual and proprioceptive systems [13]. With the impairment of one system, the other two have to compensate more to sustain postural control [14]. Degeneration and consecutive morphological changes in otolith organs and the vestibular epithelium can be responsible for the increasing number of older individuals suffering peripheral vestibular disorders, e.g., benign paroxysmal positional vertigo (BPPV) [15]. Postural stability is known to be decreased with visual impairment due to age-related macular degeneration [16]. Furthermore, sensorimotor deficits due to aging and a significantly increasing incidence of neurodegenerative conditions such Parkinson’s disease starting at the age of > 60 years lead to less proprioceptive input and neuromuscular control and therefore promote imbalance [17, 18]. The Bárány Society considered it necessary to attend to the phenomena of presbyvestibulopathy and developed diagnostic criteria for the manifestation of unsteadiness, gait disturbance, and falls [5].

Despite diagnostic advances, many cases of VDB do not benefit from a single medical or surgical therapy [19]. This might be especially true for older patients due to the multifactorial aetiology and the lack of obvious cause-specific pathology. So-called syndromes such as presbyvestibulopathy or presbystatis might pose the challenge of performing a multi-systemic efficient examination and are recommended to be treated symptomatically to achieve the fastest and most efficient therapy possible [20]. It is well established that older patients with VDB benefit from physical therapy that addresses consequences such as imbalance and falls and is unspecific in regard to and independent of the underlying pathology [21].

Whitney et al. [22] describe key interventions of physical therapy for patients experiencing VDB. Vertigo occurring at change of position, similarly to BPPV, can be treated by canal repositioning manoeuvres (CRMs). Dizziness with head movements caused by visual blurring requires exercises for adaption of the vestibule-ocular reflex with complex backgrounds. Despite the aetiology and especially when patients have problems with balance during standing or walking, experts recommend additional balance exercises. Patient education can be useful for phobic components of dizziness or fear of falling [22]. Especially for multifactorial VDB in older individuals, a customized and problem-oriented approach is recommended to identify key symptoms and priorities of individualized rehabilitation to promote general mobility and participation [23, 24]. Evidence-based physical therapy options have increased in recent years, whereas new interventions, e.g., virtual reality, have broadened the perspectives of physical therapists [22]. In older patients > 65 years with VDB, this systematic review aims to provide an overview of the effects of physical therapy interventions, including adverse effects, that address mobility and participation in and additionally, to characterize the used primary and secondary outcomes according the International Classification of Functioning, Disability and Health (ICF).

## Methods

Reporting of this review was guided by the Preferred Reporting Items for Systematic Reviews and Meta-Analyses (PRISMA) checklist [25] and the reporting guideline Synthesis Without Meta-analysis (SWiM) in systematic reviews [26]. The study protocol was registered at PROSPERO (18th of December 2017) and can be accessed at (http://www.crd.york.ac.uk/PROSPERO/display_record.php?ID=CRD42017080291) with registration number PROSPERO 2017 CRD42017080291.

### Identification of studies

The development of the search strategy followed the PICOS scheme and the Cochrane Handbook for Systematic Reviews of Interventions 6.0 [27]. In brief, we combined the characteristics of the target population and variations in the spelling of “physical therapy”. For details, see Table 1**.** As described, literature strongly recommends to consider and treat VDB in older adults as multifactorial. Therefore, we decided to include a wide range of aetiologies and physical therapy interventions of papers into our review. We applied the following inclusion criteria:

1. The population of the included studies had a mean age of ≥65 years in the intervention or control group or were described as a subgroup that experienced vertigo, dizziness or balance disorders.
2. Intervention was defined as all kinds of physical therapy and related intervention components also included as a subgroup.
3. All study designs with control group designs, such as individually randomized, cluster-randomized and non-randomized controlled trials were included. Systematic reviews and meta-analyses were included to be used as source for backward citation tracing.
4. The studies were carried out between 2007 and 2019.
5. Language was German or English.

**Table 1:** Search strategy for MEDLINE via PubMed

No.	Search terms
1	“Labyrinth Diseases”[MeSH]
2	“Dizziness”[MeSH]
3	“Vestibule, Labyrinth”[MeSH]
4	“Vestibulocochlear Nerve Diseases”[MeSH]
5	vertig*[Title/Abstract]
6	dizz*[Title/Abstract]
7	1 OR 2 OR 3 OR 4 OR 5 OR 6
8	“Physical Therapy Modalities”[MeSH]
9	“Physical Therapists”[MeSH]
10	“Physical Therapy Specialty”[MeSH]
11	“Exercise”[MeSH]
12	physiotherap*[Title/Abstract]
13	physical therap*[Title/Abstract]
14	balanc* train*[Title/Abstract]
15	vestibul* rehabilitat*[Title/Abstract]
16	8 OR 9 OR 10 OR 11 OR 12 OR 13 OR 14 OR 15
17	7 AND 16
	Publication date 2007–2017

We excluded studies with healthy adults, as well as with persons with no or insufficient description of age. We also excluded surgical or pharmacological interventions.

An initial systematic search of the literature was conducted in MEDLINE (via PubMed), Cochrane Library, CINAHL and PEDro and took place on the 27th of November 2017. A search update followed on the 16th of July 2019. Additional sources were identified between November 2017 and April 2018 and between July and August 2019 by searching the World Wide Web, reference lists of included studies and the Bárány Society congress papers of 2010, 2014, 2016 and 2018. Search strategies for PubMed are shown in Table 1, strategies for all other databases are shown in Additional file 1.

### Study selection

We managed records identified from database searching by Covidence software (https://www.covidence.org/). Additional citations from other sources were handled manually. Deduplication of database records was done with Covidence. Based on the predefined inclusion criteria, two independent authors (VR and ES) screened titles and abstracts and removed irrelevant studies. Detailed reasons for exclusion were documented.

### Data extraction and critical appraisal

Two independent reviewers (VR and PB) extracted data using a template for the intervention description and assessed the methodological quality of eight (38%) studies in duplicate and 13 (62%) studies for feasibility reasons by VR following the risk-of-bias assessment of Cochrane handbook 5.1.0 [27] and using RevMan 5.3 software [28] to generate graphs. Disagreement was resolved by discussion and consensus or by consulting a third reviewer (MM), if required. The data extraction sheet is available from the authors on request.

### Data synthesis

The included studies were mostly heterogeneous in terms of interventions and outcome measures. Therefore, we used inductive categories for grouping by interventions, comparisons and by outcomes. As expected, a narrative synthesis across all types of interventions was conducted respecting all outcome measures covering aspects of World Health Organization’s (WHO) model of the International Classification of Functioning, Disability and Health (ICF), quality of life and general health. Mean or median differences (MD) between groups at last follow-up were used or calculated to define the change direction (advantage, no difference or unclear). A meta-analysis was not possible due to insufficiently or heterogeneous reported data [29]. Harvest plots were used for summarizing data and visualization of distinct interventions compared to no/sham intervention or to usual care. The guidelines for Grading of Recommendations Assessment, Development and Evaluation (GRADE) were used to rate certainty of findings for each outcome and were carried out in duplicate (VR and ES). We report the effects of the interventions on the primary outcome (if specified) and summarize the direction of the effects on secondary outcomes.

## Results

**Fig. 1:**
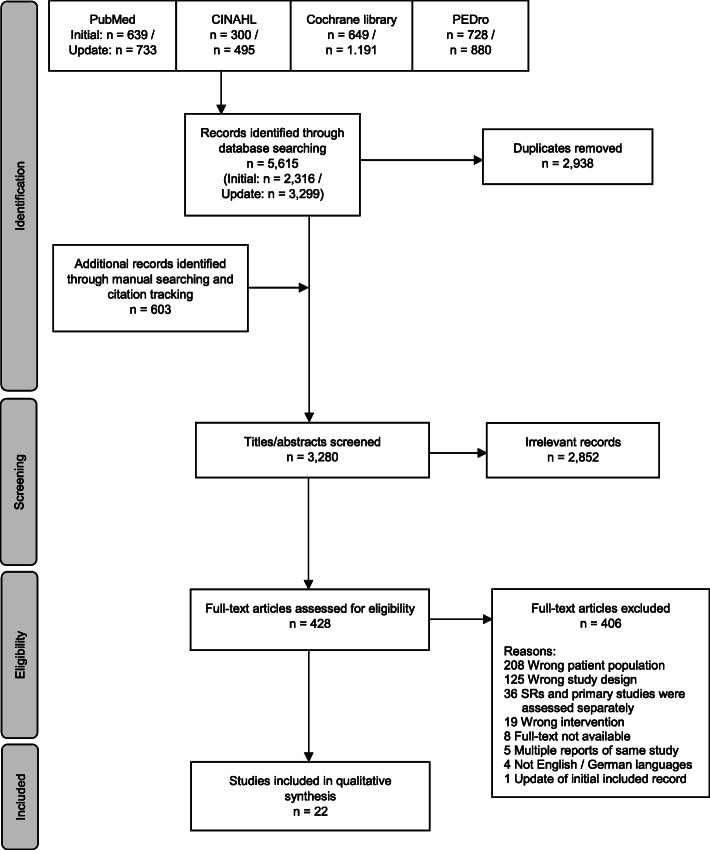
PRISMA flowchart

In the initial search, we identified 2316 records, and the search update revealed 3299 records through database searching. Additional 603 papers were identified through manual searching and from backwards citation tracing from identified systematic reviews. After deduplication, 3280 titles and abstracts were screened. Full texts of 428 studies were screened. The screening process is shown in Fig. 1.

The sample of the initial literature search comprised 16 studies [30–45], the search update revealed 5 additional studies [46–50], and one study that was updated due to a new follow-up publication [51]. Thus, the final sample comprised 22 studies with 1876 participants. All studies but two were randomized controlled trials. The latter were non-randomized controlled trials [36, 50].

### Setting and participant characteristics

The studies were conducted in 14 countries between 2008 and 2018 and took place in hospitals (7 studies), primary care (medical or physical therapy) practices (3 studies) or outpatient clinics (of a university) (6 studies) residential homes (1 study), at home (2 studies). The setting of three studies was not described. The mean age of the participants in the total population ranged from 60.0 to 85.5 years, since we also included studies in which either the intervention or control had a mean age of ≥65 years of age, and symptoms of VDB varied from cardinal symptoms of dizziness (4 studies), balance disorder (3 studies) and general vestibular dysfunction (1 study) to a specific underlying pathology such as Parkinson’s disease (4 studies), benign paroxysmal positional vertigo (BPPV) (2 studies), stroke (2 studies), fall-related conditions (2 studies), visual impairment (1 study) or cervicogenic dizziness (1 study). A table listing the characteristics of subjects is shown in Additional file 2.

### Interventions and comparisons

Interventions included unspecified vestibular rehabilitation (VR) (8 studies), specific programmes (e.g., Cawthorne-Cooksey or Otago) (4 studies) and other special forms of (vestibular) exercise therapy such as computer-assisted training (CAVR) (5 studies), Tai Chi (TCVR) (3 studies), canal repositioning manoeuvres (CRMs) (1 study) and manual therapy (1 study). Interventions were compared to usual care, no/sham interventions or to other interventions (e.g. variations of an established programme). A table listing the intervention and control interventions is shown in Additional file 2.

### Risk of bias of included studies

**Fig. 2:**
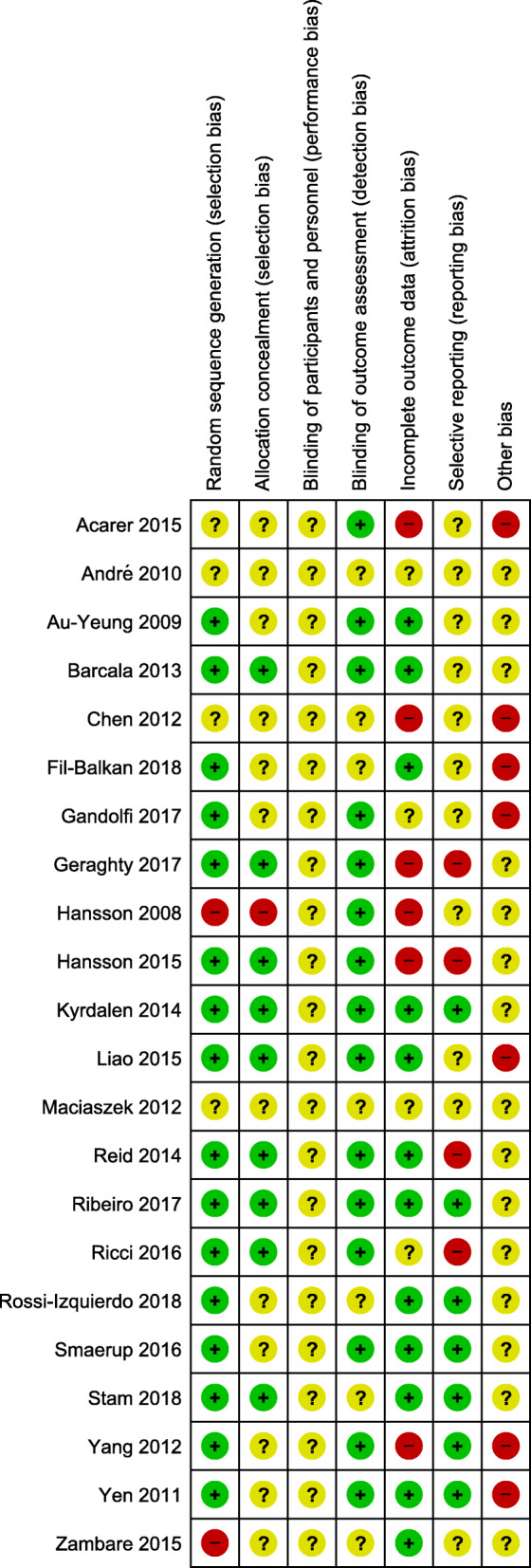
Risk of bias within included studies

**Fig. 3:**
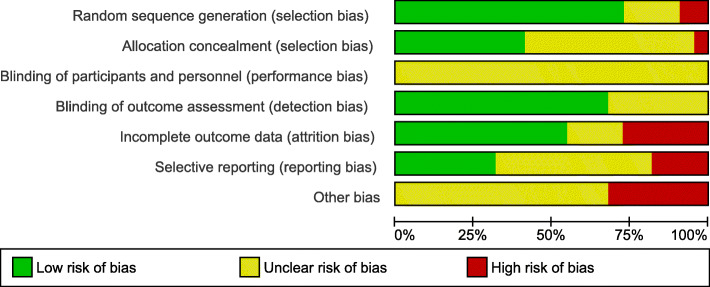
Risk of bias across included studies

The risk-of-bias assessment revealed varying methodological quality/ internal validity. Details are shown in Fig. 2. Detailed descriptions of assessment are given in supplementary data (s. Additional file 3). The risk of bias across studies is shown in Fig. 3.

### Outcome measures

Reported outcome measures among the 22 included studies, varied largely. Sixteen studies investigated static or dynamic balance or postural control. Aspects of mobility, e.g., walking ability, functional mobility or activity level, were assessed in 9 studies. Dizziness symptoms, such as frequency, intensity or its impact, were addressed in 8 studies. Six studies carried out an assessment of (risk of) falls, and 5 studies addressed quality of life. Four studies reported lower extremity muscle strength, 2 analysed proprioception, and some single studies evaluated various self-perceived outcomes. Primary outcome(s) were stated in the half of all included studies (11 studies). When attributed to ICF components, 4 studies assessed body functions and structures, 5 activities and participation and 2 both components. Primary and secondary outcomes measures are shown in Additional file 4.

#### Effects of interventions

Summary of study results are given in Additional file 5. Additional file 6 includes harvest plots summarizing the effects of included studies. The quality of evidence and summary of findings for each outcome is shown in a detailed table in Additional file 7.

### Canal repositioning manoeuvres

#### CRM versus CRM variations

Comparing CRM (Epley manoeuvre) with CRM and distinct additional instructions like wearing a neck collar for 48 h after manoeuvre or using a mini-vibrator placed on the mastoid of affected side during manoeuvre revealed no advantage for posterior canal BPPV caused by canalolithiasis as measured by the Dizziness Handicap Inventory (DHI) (no primary outcome stated, *n* = 53 participants) [31].

##### Vestibular rehabilitation

A total of 12 studies (55%) with 1284 participants investigated vestibular rehabilitation (VR) [30, 35–37, 41, 42, 44, 47–51] which was therefore the most investigated intervention.

#### VR compared to usual care

The comparison of internet-based VR and usual care showed an effect on Vertigo Symptom Scale (VSS-SF) total score (primary outcome) in favour of VR (*n* = 296 participants, − 2.26 points, *p* = .02) for patients with dizziness over the last 2 years and still experiencing dizziness triggered by head movements. Secondary outcomes showed mixed effects. Analysis of DHI score (− 5.58 points, *p* = .01), and the patient reported improvement (*p* < .001) revealed effects in favour of VR. No significant differences were found in the Hospital Anxiety and Depression Scale (HADS) [35].

No difference in DHI (primary outcome) was reported for patients with dizziness when comparing usual care with a multicomponent program that includes the assessment of fall-risk increasing drugs (FRIDs) stepped mental health care or exercise therapy (*n* = 168 participants) in a RCT. No difference of dizziness frequency, presence of anxiety and depressive disorder, QoL and fall frequency was found [49].

A RCT with 165 participants experiencing balance disorders compared the Otago programme with receiving a fall-prevention booklet and continuing usual activities (optimized usual care). No effect in favour of the intervention could be observed in the primary outcomes mCTSIB, Limits of Stability (LOS), Rhythmic Weight Shift (RWS). Within secondary outcomes, an effect in favour of intervention was shown in the step test (worse leg) (+ 2.10 steps/15 s, *p* ≤ .001), in hip abductor muscle strength (+ .02 kg/kg, *p* ≤ .001), in the Walk-across Test (WA) (− 2.17 cm, *p* ≤ .001), in the Functional Reach Test (FRT) (+ 2.95 cm, *p* ≤ .001) and on the Human Activity Profile–Adjusted Activity Score (HAP-AAS) (+ 4.57 points, *p* ≤ .001). No effects were reported regarding Sit to Stand Test (STS), the Five Times Sit to Stand Test (5x-STS), muscle strength of quadriceps and dorsiflexors, walking speed, the Step Quick Turn test (SQT), in quality of life as measured by Assessment of Quality of Life (AQoL) and falls measured in the Modified Falls Efficacy Scale (MFES) [44].

A non-randomized study with 60 participants experiencing balance disorders and a history of falls or having fear of falling investigated additional Cawthorne-Cooksey exercise programme versus conventional physical therapy did not specify a primary outcome. An effect in favour of the intervention (− 0.77 points, *p* = .030) as measured by the Visual Analogue Scale of Fear of Falling (VAS-FOF) and in the Dynamic Gait Index (DGI) (+ 1.3 points, *p* = .013) was reported. No differences in Berg Balance Scale (BBS) and the likelihood of falls were found [50].

A RCT with 660 participants with mild to moderate Parkinson’s Disease (Hoehn and Yahr stages 2–3) evaluated the effectiveness of VR versus usual care. The study did not specify a primary outcome**.** Mixed results were found: A significant benefit of + 9 points (*p* = < .05) on BBS, + 4 points (*p* = < .05) in DGI and + 27.5 points for Activities-specific Balance Confidence (ABC) (*p* < .05). No significant difference was found in mCTSIB total score, Unified Parkinson’s Disease Rating Scale (UPDRS), Timed-Up and Go test (TUG) and Quality of life measured by the Parkinson’s Disease Questionnaire (PDQ-39) [30].

When comparing classical physiotherapy (described as “individually tailored and including flexibility, strengthening, posture, breathing balance, walking exercises, and other functional activities”) with additional sensorimotor integration training versus classical physiotherapy (*n* = 30 participants with Parkinson’s Disease Hoehn and Yahr stages 2–3, no primary outcome stated), mixed results were found in a RCT. Effects in favour of intervention were found in the 5th position (+ 24.16, *p* = .027) and composite (+ 12.8, *p* = .042) of Computerized Dynamic Posturography – Sensory Organization Test (CDP-SOT) and in vestibular system score (VEST) in Computerized Dynamic Posturography –Sensory Analysis (CDP-Sensory) (+ 25.43, *p* = .048), on BBS (+ 10.34 points, *p* = .037) and in TUG (− 4.11 s, *p* = .002). No differences were reported for 6th position of CDP-SOT, somatosensory system score (SOM), visual system score (VIS) and visual preference score (PREF) in CDP-Sensory, Unified Parkinson’s Disease Rating Scale (UPDRS) and the Functional Reach Test (FRT) [47].

Moderate quality of evidence exists, that VR is superior to usual care to improve VDB symptoms, balance and mobility, but not postural control, the impact of VDB on ADL and the presence of anxiety and depression, Parkinson’s disease specific ADL, quality of life, frequency of falls and fear of falling.

#### VR versus no intervention

Two studies investigated VR versus no intervention.

A RCT (*n* = 85 participants with fall-related wrist fractures) showed no differences in primary outcomes (tandem standing with eyes open and closed and walking in a modified figure of eight). In secondary outcomes, no differences were reported when measuring SOLEO, SOLEC, 5x-STS, postural sway, vibration sense, head-shake test, EQ. 5D-VAS and walking variations [37].

A non-randomized study (*n* = 58 participants with multisensory dizziness) stated no primary outcome. Mixed effects were found. An improvement in standing on one leg with eyes closed (SOLEC) (+ 1 s, *p* = .038) and in walking heel to toe (− 2 steps, *p* = .044). No difference was observed in standing on one leg with eyes open (SOLEO), tandem standing with eyes open and closed, DHI, steps outside during walking in a figure of eight and the risk of falls maintained [36].

Training computer dynamic posturography exercises compared to no intervention (*n* = 139 participants experiencing balance impairment without a vestibular disease, no primary outcome stated) revealed to no differences in SOT, LOS, DHI, TUG and FES-I in a four-arm study, for which other comparison groups are described as follows [51].

#### New variations versus established forms of VR

VR in addition to CRM was compared to the CRM alone (*n* = 16 participants with BPPV for at least 6 months) in a RCT. Primary outcomes showed mixed effects: A difference in Maximum Excursion (MXE) of LOS (+ 17%, *p* < .05) and DGI (+ 4 points, *p* = .05) in favour of intervention and no differences in mCTSIB and movement velocity (MVL) of LOS. Secondary outcomes also revealed mixed results: a difference in tandem end sway (1 s in the *p* < .05) favouring intervention and no difference in sway in Unilateral Stance Test (US) and VAS [41].

A RCT with 125 participants (older people referred to a Falls Outpatient Clinic) investigated the Otago exercise programme in groups compared to the Otago exercise programme at home. The primary outcome BBS showed no difference. Secondary outcomes revealed mixed effects. Significant differences in 5x-STS (+ 2.2 s, *p* = .005) and TUG (− 2.4 s, *p* = .038) were reported. No differences were shown in quality of life measuring the short-form questionnaire SF-36 and on the Fall Efficiency Scale International (FES-I) [48].

A RCT with 82 participants with dizziness resulting from a vestibular disorder assessed a multimodal version of the Cawthorne-Cooksey programme versus the conventional version and observed no difference in primary outcome DGI. Also secondary outcomes showed no difference measuring STS, Romberg, tandem stand, sensorial, unipedal and handgrip strength, TUG, multidirectional FRT and fall rate [42].

A four-arm RCT compared VR with computer dynamic posturography exercises to exposure to optokinetic stimuli and exercises at home based on the Cawthorne-Cooksey programme in patients with balance impairment without a vestibular disease. Information about changes in SOT, DHI, TUG and FES-I is missing. No primary outcome was stated [51].

Moderate quality of evidence exists, that VR in addition to CRM is superior to CRM alone to improve balance. Very low quality of evidence exists, that the Otago exercise programme in groups is superior to the Otago exercise programme at home to improve lower extremity strength and mobility.

#### Computer-assisted VR

Five studies investigated computer-assisted VR (CAVR) (237 participants) [34, 38, 43, 45, 46].

#### CAVR versus usual care

No information about the comparison between WiiFit training and traditional exercises (*n* = 36 participants with idiopathic Parkinson’s Disease Hoehn and Yahr stages 2–3) is provided, but the comparison of virtual reality-based Wii Fit training with subsequent treadmill training to fall-prevention education with no structured programmeis described. This third arm of the RCT is described hereafter [38]. A RCT with 20 participants with chronic stroke-related complaints investigated additional balance training using the Wii Fit programme to conventional physical therapy in comparison to conventional physiotherapy. No primary outcome was stated. No difference was reported in balance, body symmetry, BBS, TUG and 7-level functional independence measure (FIM) [46].

#### CAVR versus no intervention

Neither effects in SOT nor in the Verbal Reaction Time (VRT) were found when virtual reality-augmented balance training with PT were compared with no intervention (*n* = 42 participants with Parkinson’s disease Hoehn and Yahr stages 2–3). No primary outcome was stated [45].

#### CAVR versus other interventions

A three-arm RCT (n = 36 participants) explored virtual reality-based Wii Fit training with subsequent treadmill training in comparison to fall-prevention education with no structured programme for idiopathic Parkinson’s Disease (Hoehn and Yahr stages 2–3). No primary outcome was stated. Mixed results were found. Advantages in gait parameters (+ 12.87 cm/s, *p* < .05) in regard to velocity, (+ 15.41 cm, *p* < .05) stride length, (+ 16.5 N,, *p* < .05) hip flexors, (+ 12.5 N, *p* < .05) hip extensors, (+ 14.6 N, *p* < .05) knee flexors, (+ 28.1 N, *p* < .05) knee extensors, (+ 37.5 N, *p* < .05) ankle dorsiflexors and (+ 25.5 N, *p* < .05) ankle plantar flexors, as well as (+ 20.5, *p* < .05) in vestibular ratio of SOT. Also a significant difference (+ 4.59 points, *p* < .05) in the Functional Gait Assessment (FGA) was observed. As the third arm, when the traditional exercise group (CG) was compared with the fall-prevention education group (CoG), all parameters changed significantly in the last follow-up except for the vision component of SOT. No primary outcome was stated. Changes in general were greater when WiiFit was compared with fall-prevention education than when traditional exercises were compared with education [38].

Home exercises supported by the “Move it to improve it” (Mitii) computer programme versus a printed home programme (*n* = 63 participants with vestibular dysfunction) showed no difference in the primary outcome one-leg stand test. No difference in secondary outcomes Motion Sensitivity, VAS, Chair stand test, DHI, DGI, quality of life measured with SF-12 [43].

A RCT compared in-home virtual reality balance training (TeleWii) to in-clinic sensory integration balance training (*n* = 76 participants with Parkinson’s Disease modified Hoehn and Yahr stages 2.5–3). No significant differences in the primary outcome BBS as well as ABC, 10-MW, DGI, Quality of life measuring PDQ-39, and in falls were observed [34].

Moderate quality of evidence exists, that virtual reality-based Wii Fit training with subsequent treadmill training is superior in comparison to fall-prevention education with no structured programme to improve gait parameters, lower extremity strength, balance and to improve postural control.

##### Tai chi as VR

Tai Chi as a form of VR was used in three studies (216 participants) [32, 33, 39].

#### Tai chi VR (TCVR) versus no/sham intervention

A RCT (*n* = 40 participants experiencing dizziness within the past year, no primary outcome stated) investigated TCVR compared to no intervention and reported significant differences (+ 61 cm, *p* = .050) forward, (+ 1.37 cm, *p* = .024) backward, (+ 28.57 cm^2^, *p* = .002) in the maximal sway area of the LOS and (− .23 s, *p* = .003) in the 8-ft up-and-go test. No differences were found in right- and leftward of LOS [39].

TCVR was compared to music percussion as sham intervention (n = 40 participants with visual impairment) in a RCT where no primary outcome was stated. Mixed effects were found. Differences (− 30.1%, *p* = .032) of the absolute angle error of the passive knee joint repositioning test, (+ 59.7%, *p* = .006) in the visual ratio and (+ 50.3%, *p* = .048) in the vestibular ratio of SOT. No difference were reported in concentric isokinetic knee extensor or flexor strength of dominant leg and in somatosensory ratio [33].

Low quality of evidence exists, that TCVR is superior to no/sham intervention to improve postural control. Very low quality of evidence exists, that TCVR is superior to no/sham intervention to improve mobility.

#### TCVR versus other interventions

A RCT with 136 participants with a history of stroke at least 6 months ago exploring TCVR in comparison to breathing and stretching exercises reported mixed effects in primary outcomes LOS and SOT: A difference (*p* = .005) in reaction time of non-affected side, (*p* = .005) in end-point excursion of non-affected and affected sides and (*p* = .05) backwards and forwards in LOS. No difference in all conditions of equilibrium score and sensory ratios of SOT and in reaction time of all other sides in LOS. Secondary outcome TUG showed no difference [32].

Very low quality of evidence exists, that TCVR is superior to breathing and stretching exercises.

##### Manual therapy

Manual therapy using Sustained Natural Apophyseal Glides (SNAGs) was investigated in one RCT (*n* = 86 participants with cervicogenic dizziness), which reported significant effects in primary outcome (− 18.4 mm, *p* = .01) on visual analogue scale (VAS) dizziness intensity compared to the sham intervention. Secondary outcomes revealed mixed effects. Effects (− 0.9 points, *p* < .001) in dizziness frequency, no differences in VAS pain and DHI. In the same study, using Maitland mobilizations instead of SNAGs, Maitland mobilizations did not change the primary outcome VAS dizziness intensity. Secondary outcomes showed mixed effects: Effects (− 7.6 points, *p* = .04) in DHI and no effects in dizziness frequency and VAS pain [40]. Very low quality of evidence exists, that SNAGs are superior to sham intervention, but equal to Maitland mobilizations to improve dizziness intensity. Maitland mobilizations are with very low quality of evidence superior to SNAGs and sham intervention to improve the impact of VDB on ADL.

#### Adverse effects

Mild transient pain in the lower cervical spine or upper arm 24 h after SNAGs was reported [40]. No other adverse effects were reported.

## Discussion

### Main findings

This review identified the quality of evidence of studies investigating physical therapy treatment options for older people with VDB addressing mobility and participation.

Studies were heterogeneous in terms of internal validity and used outcome measures. VR, VR in addition to CRM and MT showed beneficial effects [35, 40, 41], even though the quality of evidence is rather low.

CRMs for the treatment of vertigo in adults are well researched [52] and successfully practised, but the included studies with older patients show that high-quality evidence is scarce. We identified only one study investigating Epley manoeuvre in variations for older adults [31]. Considering BPPV as the most relevant diagnosis for dizziness [53] and the increasing number of older individuals suffering BPPV across their lifespan [15], findings of this review detect a lack of evidence, if CRMs are equally effective for older individuals in particular. When VR training is applied in addition to CRM in older adults, moderate quality of evidence guides to a benefit in balance [41], which is also obviously clinically relevant [54]. Results in functional outcomes emphasize the findings of a Cochrane review [52] indicating that VR including gait and balance training is recommended regardless of aetiology and as an addition to CRM for long-term functional effects.

VR is as effective as usual care when exercise therapy has an optional character in regard to impact of dizziness on ADL [49]. Thus, our findings underline that exercise therapy has to play a central role in treatment of VDB to support vestibular compensation [55]. VR is also as effective as no intervention in a study [37] when the investigated patients are a specific subgroup with a fall-related wrist fracture and generalisation of the results might be done with care. A further aspect relevant to clinical decisions is that more people in the VR group refrained from participation, so one might conclude that VR group training is less acceptable. Variations of established Otago-programme were not superior to the programme itself [48]. Effects pre- to post-treatment in both groups show the effectiveness of the Otago exercise programme on balance, mobility, quality of life and falls independently of whether delivered in the group session or, as usual at home. A recent systematic review including patients aged from 60 to 95 years confirms the effectiveness of the Otago exercise programme in every variation for an improvement in balance and functional ability [56].

Training with a computer might be less motivating without a personal supervision and so not superior to a leaflet with exercises. Especially for older individuals living alone in the community, a supervising PT might be a relevant social contact. Tele-rehabilitation, e.g. via skype, treating other health conditions in other countries report positive experiences from both PTs and patients [57]. Supervised training compared to unsupervised was shown to be more effective even for healthy older adults in other studies [58]. Unless social aspects, there might be an additional benefit by the enrichment of environment on visual or vestibular-ocular issues than only balance or VR training, but not on functional outcomes.

Our review showed that Tai Chi is not effective to improve postural control in older persons with VDB [32]. In contrast to that, a Cochrane review investigating fall prevention interventions underscored the potential of Tai Chi to significantly reduce risk of falling for older people living in the community [59] and also the psychosocial effects of Tai Chi on nursing home residents on quality of life, especially in mental components were shown [60]. Our study investigated patients having VDB after a stroke, the Cochrane review older adults solely with VDB. Thus, Tai Chi seems effective for VDB, but not for VDB after a stroke.

In contrast to frequent recommendations to patients with VDB, manual therapy revealed not to be effective in our target population [40]. Considering other literature and the likelihood of injuries following cervical manipulations is increased with age-related diseases like osteoporosis or long-term use of anticoagulant therapy [61] and specific red flags should be assessed before using manual interventions in elderly [62]. Considering the debatable entity of cervicogenic dizziness [63], MT cannot be recommended for older adults. Combinations of VR with MT are already shown as synergistic in few case series with young patients [64], but further research is needed [65].

### Limitations

Due to the heterogeneity of interventions, outcomes and study population, comparison of the findings was difficult. We identified many studies investigating physical therapy for VDB, but such investigations frequently had designs without control groups and were conducted in a younger population, what is especially reflected in regard to CRMs. With regard to the methodological quality of the included studies, randomization process, allocation concealment, blinding of participants and personnel were not reported sufficiently. Blinding of outcome assessors as well as drop-outs were described in several studies, but the number of withdrawals frequently was unbalanced between groups. However, we are confident, that our review added reasonable knowledge relevant to clinical decision making because its rigorous quality assessment.

Due to the restriction of the earliest publication year to 2007 and the language to English or German, some meaningful articles published before 2007 or in other languages might have been excluded. Primary outcome was clearly stated only in 7 studies [34, 35, 40, 42, 43, 48, 49]. Descriptions of more than one single primary outcome were reported in 4 studies [32, 37, 41, 44]. In regard to the GRADE approach, overall risk of bias, indirectness, inconsistency, imprecision and publication bias were assessed, whereas imprecision was ranked down for every study due to a narrative synthesis in this review.

## Conclusions

Vestibular rehabilitation in any variation seems to be effective in treating older adults with VDB. The same applied to VR in addition to CRMs. Tai Chi and manual therapy did not show any additional benefit in comparison to usual care in an older population. Overall, quality of evidence is rather low, especially in regard to bias. Many physical therapy intervention studies focus on surrogate markers, such as specific postural or muscle functions than on patient relevant outcomes that reflect mobility and participation. In addition, there is a lack of transparent reporting of PT interventions as complex interventions what makes it hard to implement findings into day to day care. High-quality randomized trials need to be carried out in future to inform clinical decision making for the highly vulnerable group of older patients with VDB.

## Supplementary Information

**Additional file 1.** Search terms and records.

**Additional file 2.** Characteristics of included studies.

**Additional file 3.** Methodological quality of included studies.

**Additional file 4.** Primary and secondary outcome measures.

**Additional file 5.** Summary of study results.

**Additional file 6.** Harvest plots summarizing effects of interventions.

**Additional file 7.** Evidence tables rating quality of evidence and summary of findings.

## Data Availability

Data extraction sheets and further information are available from the authors by request.

## Abbreviations

10-MW: Ten meter walking
5x-STS: Five times sit to stand test
ABC: Activities-specific balance confidence
AQoL: Assessment of quality of life
BBS: Berg balance scale
CAVR: Computer-assisted vestibular rehabilitation
CDP-Sensory: Computerized dynamic posturography – sensory analysis
CDP-SOT: Computerized dynamic posturography – Sensory organization test
CG: Control group
CI: Confidence interval
CoG: Comparison group
CRM: Canal repositioning manoeuvre
DGI: Dynamic gait index
DHI: Dizziness handicap inventory
EQ 5D-VAS: Visual analogue scale of five dimensions EuroQol
FES-I: 7 item falls efficacy scale international
FGA: Functional gait assessment
FIM: 7-level functional independence measure
FRIDs: Fall-risk-increasing drugs
FRT: Functional reach test
GAD-7: Generalised anxiety disorder assessment 7 subscale
HADS: Hospital anxiety and depression scale
HAP-AAS: Human activity profile–adjusted activity score
IG: Intervention group
LOS: Limits of stability
mCTSIB: Modified clinical test of sensory interaction on balance
MFES: Modified falls efficacy scale
MT: Manual therapy
Multidirectional FR: Multidirectional functional reach
MVL: Movement velocity
MXE: Maximum excursion
PDQ-39: Parkinson's Disease Questionnaire
PHQ-9: Patient health questionnaire-9
PREF: Visual preference score
QoL: Quality of life
RWS: Rhythmic weight shift
SF-12: 12 item short form assessment of quality of life
SF-36 MH: 36 item short form assessment health-related quality of life mental health
SF-36 PH: 36 item short form assessment health-related quality of life physical health
SNAGs: Sustained natural apophyseal glides
SOLEC: Standing on one leg with eyes closed
SOLEO: Standing on one leg with eyes open
SOM: Somatosensory system score
SOT: Sensory organization test
SQT: Step quick turn test
STS: Sit to stand
TCVR: Tai Chi as vestibular rehabilitation
TUG: Timed up and go test
UPDRS: Unified Parkinson´s disease rating scale
US: Unilateral stance test
VAS: Visual analogue scale
VAS-FOF: Visual analogue scale for fear of fall
VEST: Vestibular system score
VIS: Visual system score
VR: Vestibular rehabilitation
VRT: verbal reaction time
vs.: versus
VSS-SF: Short form of vertigo symptom scale
WA: Walk across test
