# Supplementary material for: Physical therapy interventions for older people with vertigo, dizziness and balance disorders addressing mobility and participation: a systematic review
Source: BMC Geriatr. 2020 Nov 23;20:494. doi: 10.1186/s12877-020-01899-9 (PMC7684969; doi:10.1186/s12877-020-01899-9)
Supplement: Supplementary file 1 — Additional file 1. Search terms and records. [file 12877_2020_1899_MOESM1_ESM.docx]

**Additional file 1** Search terms and records

MEDLINE via PubMed

| **No.** | **Search terms** | **Results**  **(23.11.2017)** | **Results**  **(16.07.2019)** |
| --- | --- | --- | --- |
| 1 | "Labyrinth Diseases"[Mesh] | 22.720 | 23.834 |
| 2 | "Dizziness"[Mesh] | 4.602 | 5.012 |
| 3 | "Vestibule, Labyrinth"[Mesh] | 16.549 | 17.124 |
| 4 | "Vestibulocochlear Nerve Diseases"[Mesh] | 8.882 | 9.357 |
| 5 | vertig*[Title/Abstract] | 12.820 | 14.035 |
| 6 | dizz*[Title/Abstract] | 16.119 | 17.928 |
| 7 | 1 OR 2 OR 3 OR 4 OR 5 OR 6 | 61.543 | 65.491 |
| 8 | "Physical Therapy Modalities"[Mesh] | 131.579 | 144.596 |
| 9 | "Physical Therapists"[Mesh] | 1.045 | 1.520 |
| 10 | "Physical Therapy Specialty"[Mesh] | 2.536 | 2.719 |
| 11 | "Exercise"[Mesh] | 156.856 | 180.608 |
| 12 | physiotherap*[Title/Abstract] | 21.117 | 24.090 |
| 13 | physical therap*[Title/Abstract] | 20.814 | 23.575 |
| 14 | balanc* train*[Title/Abstract] | 11.208 | 13.354 |
| 15 | vestibul* rehabilitat*[Title/Abstract] | 1.352 | 1.610 |
| 16 | 8 OR 9 OR 10 OR 11 OR 12 OR 13 OR 14 OR 15 | 8.718 | 10.402 |
| 17 | 7 AND 16 | 919 | 1.079 |
|  | Publication date | 2007-2017:  639 | 2007-2019:  733 |

CINAHL

| **No.** | **Search terms** | **Results**  **(23.11.2017)** | **Results**  **(16.07.2019)** |
| --- | --- | --- | --- |
| 1 | (MH "Labyrinth Diseases") | 346 | 589 |
| 2 | (MH "Dizziness") | 1.356 | 2.254 |
| 3 | (MH "Vestibule, Labyrinth") | 693 | 1.124 |
| 4 | (MH "Vestibulocochlear Nerve Diseases") | 158 | 294 |
| 5 | vertig* | 2.415 | 3.849 |
| 6 | dizz* | 3.471 | 5.755 |
| 7 | 1 OR 2 OR 3 OR 4 OR 5 OR 6 | 6.062 | 9.965 |
| 8 | (MH "Physical Therapy") | 23.309 | 31.222 |
| 9 | (MH "Physical Therapists") | 7.641 | 10.446 |
| 10 | (MH "Exercise") | 27.155 | 44.487 |
| 11 | physiotherap* | 14.432 | 20.533 |
| 12 | physical therap* | 46.737 | 61.736 |
| 13 | balanc* train* | 1.631 | 2.959 |
| 14 | vestibul* rehabilitat* | 348 | 521 |
| 15 | 8 OR 9 OR 10 OR 11 OR 12 OR 13 OR 14 | 79.649 | 115.332 |
| 16 | 7 AND 15 | 489 | 687 |
|  | Publication date | 2007-2017:  300 | 2007-2019:  495 |

Cochrane library

| **No.** | **Search terms** | **Results**  **(23.11.2017)** | **Results**  **(16.07.2019)** |
| --- | --- | --- | --- |
| 1 | MeSH descriptor: [Labyrinth Diseases] explode all trees | 652 | 705 |
| 2 | MeSH descriptor: [Dizziness] explode all trees | 622 | 676 |
| 3 | MeSH descriptor: [Vestibule, Labyrinth] explode all trees | 204 | 210 |
| 4 | MeSH descriptor: [Vestibulocochlear Nerve Diseases] explode all trees | 90 | 82 |
| 5 | vertig*:ti,ab,kw | 4.587 | 4.477 |
| 6 | dizz*:ti,ab,kw | 9.976 | 12.874 |
| 7 | 1 OR 2 OR 3 OR 4 OR 5 OR 6 | 13.913 | 16.577 |
| 8 | MeSH descriptor: [Physical Therapy Modalities] explode all trees | 22.007 | 22.904 |
| 9 | MeSH descriptor: [Physical Therapists] explode all trees | 68.128 | 96 |
| 10 | MeSH descriptor: [Physical Therapy Specialty] explode all trees | 128 | 118 |
| 11 | MeSH descriptor: [Exercise] explode all trees | 19.952 | 22.075 |
| 12 | physiotherap*:ti,ab,kw | 8.374 | 13.317 |
| 13 | physical therap*:ti,ab,kw | 26.239 | 50.876 |
| 14 | balanc* train*:ti,ab,kw | 3.261 | 6.219 |
| 15 | vestibul* rehabilitat*:ti,ab,kw | 269 | 480 |
| 16 | 8 OR 9 OR 10 OR 11 OR 12 OR 13 OR 14 OR 15 | 59.765 | 88.573 |
| 17 | 7 AND 16 | 987 | 1.453 |
|  | Publication date | 2007-2017:  649 | 2007-2019:  1.191 |

PEDro

| **No.** | **Search terms** | **Results**  **(23.11.2017)** | **Results**  **(16.07.2019)** |
| --- | --- | --- | --- |
| 1 | vertig* AND physi* | 35 | 39 |
| 2 | vertig* AND balanc* | 22 | 26 |
| 3 | vertig* AND vestibul* rehabilitat* | 43 | 48 |
| 4 | vertig* AND exercis* | 44 | 49 |
| 5 | dizz* AND physi* | 52 | 67 |
| 7 | dizz* AND balanc* | 44 | 52 |
| 8 | dizz* AND vestibul* rehabilitat* | 62 | 73 |
| 9 | dizz* AND exercis* | 66 | 85 |
| 10 | vestibul* AND physi* | 74 | 89 |
| 11 | vestibul* AND balanc* | 77 | 99 |
| 12 | vestibul* AND rehabilitat* | 115 | 136 |
| 13 | vestibul* AND exercis* | 94 | 117 |
|  |  | 728 | 880 |
