# Supplementary material for: Physical therapy interventions for older people with vertigo, dizziness and balance disorders addressing mobility and participation: a systematic review
Source: BMC Geriatr. 2020 Nov 23;20:494. doi: 10.1186/s12877-020-01899-9 (PMC7684969; doi:10.1186/s12877-020-01899-9)
Supplement: Supplementary file 2 — Additional file 2. Characteristics of included studies. [file 12877_2020_1899_MOESM2_ESM.docx]

**Additional file 2** Characteristics of included studies

| **Source/ Country** | **Design** | **Setting** | **Participants**  **n_randomized_ (n_IG_/n_C(o)G_);**  **n_follow-up_** | **Age (y),**  **Mean ± SD (Range)** | **Study Groups** | | | **Intensity of intervention** | **Observation**  **period** |
| --- | --- | --- | --- | --- | --- | --- | --- | --- | --- |
|  |  |  |  |  | **Intervention group** | **Control group(s)** | **Comparison group** |  |  |
| Acarer [30]  Turkey | RCT | Hospital and at home | 60 (30/30);  40 (29/11);  Mild to moderate Parkinson´s Disease (Hoehn and Yahr scores II-III) | IG: 67 (51–81)  CG: 60 (40–71) | Vestibular rehabilitation: Adaption, substitution, habituation and  balance exercises. Additional home exercise program | Usual care | - | 30-45 minutes  1x per week  Home exercises 30-40 minutes  2x per day  8 weeks | T0: Baseline  T1: **8 weeks** |
| André [31]  Brazil | RCT | Hospital | 53;  NN;  Posterior canal BPPV caused by canalolithiasis | 67.19 (60-91) | Epley’s manoeuver and post-manoeuver instructions (neck collar  for 48 hours; postural restrictions) | - | **CoG1:** Epley’s manoeuver **CoG2:** Epley`s maneuver and minivibrator placed on the mastoid during maneuver | **IG:** Instruction: Neck collar for 48 hours after maneuver and postural restrictions  **CoG2:** Minivibrator frequency of 92 cycles per second during maneuver | T0: Baseline  T1: when Dix-Hallpike-manoeuver is negative |
| Au-Yeung [32]  China | RCT | Various community or day care centers and at home | 136 (74/62);  108 (56/52);  History of stroke >6 months | IG: 61.7 ± 10.5  CoG: 65.9 ± 10.7 | Tai Chi and additional self-practice | - | Breathing and stretching exercises and 1 educational talk on stroke prevention | **IG/CoG:** 1 hour  1x per week  3 hours self-practice per week  12 weeks | T0: Baseline  T1: 6 weeks  T2: 12 weeks  T3: **18 weeks** |
| Barcala [46]  Brazil | RCT | Physical therapy clinic of the University | 20 (10/10);  20 (10/10);  chronic sequelae stemming from a stroke | IG: 65.2 (12.5)  CG: 63.5 (14.5) | Conventional physical therapy and additional balance training using the Wii Fit program | Usual care: Conventional physical therapy | - | **IG/CG:** 60 minutes  2x per week  5 weeks  **IG:** balance training: 30 minutes | T0: Baseline  T1: **7 weeks** |
| Chen [33]  China | RCT | Residential care homes | 40 (21/19);  23 (13/10);  Visual impairment | IG: 85.5 ± 6.9  CG: 82.9 ± 7.5 | Tai Chi | Sham intervention: Music percussion | - | **IG/CG:** 1,5 hour  3x per week  16 weeks | T0: Baseline  T1: **16 weeks** |
| Fil-Balkan [47]  Turkey | RCT | University | 30 (15/15);  15 (7/8);  Parkinson´s Disease Hoehn and Yahr stages 2–3 | IG 71.83 ± 9.71  CG 72.75 ± 9.23 | Classic physiotherapy and additional sensorimotor integration training. | Usual care: Classic physiotherapy | - | IG: 30-minute SMIT in addition  IG/CG: 1-hour per session  2x per week  6 weeks | T0: Baseline one week prior to training  T1: 6 weeks  T2: **12 weeks** |
| Gandolfi [34]  Italia | RCT | Hospital and at home | 76 (38/38);  70 (36/34);  Parkinson´s Disease  modified Hoehn and Yahr stages 2.5–3 | IG: 67.45 ± 7.18  CoG: 69.84 ± 9.41 | Remotely supervised in-home virtual reality balance training (TeleWii) | - | In-clinic sensory integration balance training | **IG/CoG:** 21 session  50 minutes per session  3x per week  7 weeks | T0: Baseline  T1: 7 weeks  T2: **11 weeks** |
| Geraghty [35]  United Kingdom | RCT | Primary care practices | 296 (160/136);  230 (112/118);  Dizziness over the last two years and still experiencing by head movements | IG: 67.3 ± 9.0  CG: 67.5 ± 11.5 | Internet-based vestibular rehabilitation | Usual care | - | **IG:** 6 sessions  6 weeks | T0: Baseline  T1: 3 months  T2: **6 months** |
| Hansson [36]  Sweden | nRCT | Physiotherapy centre | 58 (31/27);  45 (22/23);  Multisensory dizziness | IG: 80  CG: 85 | Vestibular rehabilitation | No intervention | - | 50 min per session  2x per week  9 weeks | T0: Baseline  T1: **3 months** (all)  T2: 6 months (only telephone)  T4: 9 months (only telephone)  T5: **12 months** (only telephone and DHI) |
| Hansson [37]  Sweden | RCT | Hospital | 85 (41/44);  68 (27/41);  Fall-related wrist fractures | IG: 73 ± 8  CG: 72 ± 10 | Vestibular rehabilitation  in group sessions | Not described, probably no intervention | - | IG: minutes not described  2x per week  9 weeks | T0: Baseline  T1: **3 months** |
| Kyrdalen [48]  Norway | RCT | Falls Outpatient Clinic and at home | 125 (63/62);  83 (43/40);  Older people referred to a Falls Outpatient Clinic | IG: 82.9 ± 5.0  CoG: 82.1 ± 6.4 | Group training using  the Otago exercise program | - | Home training using the Otago exercise program | IG: 45 minutes per session  2x per week  CG: 30 minutes per session  3x per week  IG / CG: Additional ≥ 3 walks of ≥ 30 minutes weekly  12 weeks | T0: Baseline  T1: 12 weeks  T2: 3 months after intervention completion  **6 months** |
| Liao [38]  China | RCT | Hospital | 36 (12/12/12);  35 (12/12/11);  Idiopathic Parkinson´s Disease (Hoehn and Yahr Stages I-III) | IG: 67.3 ± 7.1  CG: 65.1 ± 6.7  CoG: 64.6 ± 8.6 | Virtual reality based Wii Fit training with following treadmill training | Usual care: Traditional exercises with following treadmill training | Fall-prevention education | **IG/CG:** 45 minutes per session  15 minutes treadmill training  12 sessions  2x per week  6 weeks | T0: Baseline (intervention -1d)  T1: 6 weeks  (Intervention +1d)  T2: 30th day after intervention  **10 weeks** |
| Maciaszek [39]  Poland | RCT | Not described | 40 (20/20);  NN;  Dizziness within the past year | IG: 70.3 ± 5.9  CG: 69.1 ± 5.9 | Tai Chi | No intervention | - | 45 minutes  2x per week  18 weeks | T0: Baseline (intervention -1 week)  T1: **18 weeks** |
| Reid [40]  Australia | RCT | University | 86 (29/29/28);  80 (26/27/27);  Cervicogenic dizziness | IG: 60.0 ± 10.1  CG: 65.6 ± 11.0  CoG: 61.0 ± 15.7 | Mulligan SNAGs. Sustained anterior gliding and self-administered SNAGs as home exercise. | Sham intervention: Deactivated laser application to 3 sides of the neck | Maitland mobilization and range-of-motion (ROM) exercises | **IG**: 6x SNAGs (additional 6x SNAG in other direction)  Home exercise: 6x self-SNAGs  1x per day  **CoG:** 3x 30 seconds in each direction  1x per day  ROM-exercises into flexion, extension, rotation, and lateral flexion  3x per day  **CG:** with a distance of 0,5 to 1 cm  2 minutes  12 weeks | T0: Baseline  T1: 12 weeks  T2: further 12 weeks  **6 months** |
| Ribeiro [41]  Brazil | RCT | Not described | 16 (8/8);  14 (7/7);  BPPV at least 6 months | IG: Median 69 (65-78)  CoG: Median 73 (65-76) | Customized vestibular rehabilitation therapy and canalith repositioning manoeuver | - | Canalith repositioning maneuver | **IG/CoG:** CRM: up to 3x per session  (if sign persisted and Dix-Hallpike test positive)  **IG:** VR: 50 minutes per session  2x per week  13 weeks | T0: Baseline  T1: 1 week  T2: 5 weeks  T3: 9 weeks  T4: **13 weeks** |
| Ricci [42]  Brazil | RCT | Outpatient clinic | 82 (42/40);  70 (34/36);  Dizziness resulting from a vestibular disorder documented | 74 | Novel Multimodal Cawthorne & Cooksey protocol and additional | - | Conventional Cawthorne & Cooksey protocol | 50 minutes per session  2x per week  Home exercises: daily  8 weeks | T0: Baseline  T1: 8 weeks  T2: **3 months** |
| Rossi-Izquierdo [51]  Spain | RCT | Not described | 139 (35/35/34/35);  106  (27/28/30/21);  Balance impairment without a vestibular disease | IG: 76.98 ± 7.16  CG: 76.82 ± 5.74  CoG1: 74.34 ± 5.77  CoG2: 76.83 ± 6.62 | Intervention with computer dynamic posturography exercises | No intervention | **CoG1:** Intervention with exposure to optokinetic stimuli  **CoG2:** Intervention with Exercises at home based on Cawthorne–Cooksey | **IG:** 10 exercises per session  **IG/CoG1:** 1x per day to 5x per week  **CoG2:** 2x per day  **IG/CoG1/CoG2**: 15 minutes per session  2 weeks | T0: Baseline  T1: 3 weeks  T2: 6 months after VR  T3: 12 months after VR  **12,5 months** |
| Smaerup [43]  Denmark | RCT | At home | 63 (32/31);  57 (28/29);  Vestibular dysfunction | IG: 76.65 ± 7.56  CG: 78.68 ± 6.56 | Home exercises supported by the Mitii computer program | - | Printed home-training program | **IG/CoG:** 20-30 minutes per session  2x per week  combined with home exercises  1x per day  12 weeks | T0: Baseline (after end of rehabilitation in hospital)  T1: **12 weeks** |
| Stam [49]  Netherlands | RCT | Primary care practices or at home | 168 (83/85);  134 (67/67);  Dizziness | IG: 78.6 ± 7.0  CG: 79.0 ± 7.6 | (1) FRID medication adjustment in case of 3 prescribed FRIDs  (2) stepped mental health care in case of anxiety disorder and/or depression  (3) exercise therapy in case of impaired functional mobility | Usual care | - | (1) according to a list of FRIDs  (2) four subsequent steps for 6 weeks each  24 weeks  (3) 1 hour per session  2x per week  8 weeks. | T0: Baseline  T1: 3 months  T2: 6 months  T3: **12 months** |
| Yang [44]  Australia | RCT | At home | 165 (82/83);  121 (59/62);  Balance concerns | IG: 81.0 +- 5.9  CG: 80.1 +- 6.4 | Personalized, home-based exercise program (Otago) | Usual care: Usual activities and fall prevention information booklet | - | 5-8 exercises per session  20 minutes per session  5x per week  Walking program: 30 minutes  1x per day  6 months | T0: Baseline  T2: **6 months** |
| Yen [45]  Taiwan | RCT | Hospital | 42 (14/14/14);  32 (12/8/12);  Parkinson´s disease Hoehn and Yahr stages II–III | IG: 70.4 ± 6.5  CG1: 71.6 ± 5.8  CG2: 70.1 ± 6.9 | Virtual reality-augmented balance training and physical therapy | **CG1**: No intervention: Untrained  **CG2**:Usual care: Conventional balance training | - | 30 minutes per session  2x per week  6 weeks | T0: Baseline  T1: 6 weeks  T2: further 4 weeks  **10 weeks** |
| Zambare [50]  India | nRCT | Hospital | 60 (30/30);  NN;  Balance disorders and history of fall/ having fear of fall | IG: 65.03±2.55  CoG: 65.53±2.95 | Cawthorne-Cooksey exercise program with conventional physical  therapy | Usual care: Conventional physical therapy | - | 60 minutes per session  3 sessions per week  9 weeks | T0: Baseline  T1: 9 weeks  **9 weeks** |

BPPV = benign paroxysmal positional vertigo; CG = Control group; CoG = Comparison group; FRID = fall-risk-increasing drug; IG = Intervention group; Mitii = Move it to improve it; N = Number of participants; SNAGs = Sustained Natural Apophyseal Glides; RCT = randomized controlled trial; VR = Vestibular rehabilitation
