# Supplementary material for: Physical therapy interventions for older people with vertigo, dizziness and balance disorders addressing mobility and participation: a systematic review
Source: BMC Geriatr. 2020 Nov 23;20:494. doi: 10.1186/s12877-020-01899-9 (PMC7684969; doi:10.1186/s12877-020-01899-9)
Supplement: Supplementary file 3 — Additional file 3. Methodological quality of included studies. [file 12877_2020_1899_MOESM3_ESM.docx]

**Additional file 3** Methodological quality of included studies

| **Acarer 2015** |  |  |
| --- | --- | --- |
| **Bias** | **Authors' judgement** | **Support for judgement** |
| Random sequence generation (selection bias) | Unclear risk | Comment: Insufficient information. |
| Allocation concealment (selection bias) | Unclear risk | Comment: Insufficient information. |
| Blinding of participants and personnel (performance bias) | Unclear risk | Quote: Insufficient information about personnel. "The patients were actively involved in adapting the exercise program to suit their symptoms, capabilities, and lifestyles. "patients not blinded"; "patients were aware of intervention assignments" |
| Blinding of outcome assessment (detection bias) | Low risk | Quote: "The patients were assessed individually at each session, who was blinded to the patients group" |
| Incomplete outcome data (attrition bias) | High risk | Comment: Sufficient reporting of attrition with reasons, but unbalanced between groups (IG: 3%; CG: 36%). |
| Selective reporting (reporting bias) | Unclear risk | Comment: Insufficient information; No indication to study protocol. |
| Other bias | High risk | Quote: "Patients always assessed in the on-period which might have masked the under lying changes". "The lack of exercises assigned to the control group may have reduced the motivation." |

| **André 2010** |  |  |
| --- | --- | --- |
| **Bias** | **Authors' judgement** | **Support for judgement** |
| Random sequence generation (selection bias) | Unclear risk | Comment: Insufficient information. |
| Allocation concealment (selection bias) | Unclear risk | Comment: Insufficient information. |
| Blinding of participants and personnel (performance bias) | Unclear risk | Comment: Insufficient information. |
| Blinding of outcome assessment (detection bias) | Unclear risk | Comment: Insufficient information. |
| Incomplete outcome data (attrition bias) | Unclear risk | Comment: Insufficient information. |
| Selective reporting (reporting bias) | Unclear risk | Comment: Insufficient information; No indication to study protocol. |
| Other bias | Unclear risk | - |

| **Au-Yeung 2009** |  |  |
| --- | --- | --- |
| **Bias** | **Authors' judgement** | **Support for judgement** |
| Random sequence generation (selection bias) | Low risk | Quote: "aid of a computer program" |
| Allocation concealment (selection bias) | Unclear risk | Comment: Insufficient information. |
| Blinding of participants and personnel (performance bias) | Unclear risk | Comment: Insufficient information. |
| Blinding of outcome assessment (detection bias) | Low risk | Quote: "A blinded assessor examined subjects" |
| Incomplete outcome data (attrition bias) | Low risk | Comment: Sufficient reporting of attrition with reasons and quite balanced between groups (IG: 23%; CG: 16%). |
| Selective reporting (reporting bias) | Unclear risk | Comment: Insufficient information; No indication to study protocol. |
| Other bias | Unclear risk | - |

| **Barcala 2013** |  |  |
| --- | --- | --- |
| **Bias** | **Authors' judgement** | **Support for judgement** |
| Random sequence generation (selection bias) | Low risk | Quote: "Randomization numbers were generated from a randomization table at a central office." |
| Allocation concealment (selection bias) | Low risk | Quote: "A series of numbered, sealed, opaque envelopes was used to ensure confidentiality. Each envelop contained a card stipulating to which group the individual would be allocated" |
| Blinding of participants and personnel (performance bias) | Unclear risk | Comment: Insufficient information |
| Blinding of outcome assessment (detection bias) | Low risk | Quote: "evaluator who was blinded" |
| Incomplete outcome data (attrition bias) | Low risk | Comment: Sufficient information about process without withdrawals |
| Selective reporting (reporting bias) | Unclear risk | Comment: Insufficient information; No indication to study protocol. |
| Other bias | Unclear risk | - |

| **Chen 2012** |  |  |
| --- | --- | --- |
| **Bias** | **Authors' judgement** | **Support for judgement** |
| Random sequence generation (selection bias) | Unclear risk | Quote: "Stratified randomisation was used".  Comment: Insufficient information. |
| Allocation concealment (selection bias) | Unclear risk | Comment: Insufficient information. |
| Blinding of participants and personnel (performance bias) | Unclear risk | Comment: Insufficient information; Single-blind study design, but not clear if patients or personnel |
| Blinding of outcome assessment (detection bias) | Unclear risk | Comment: Insufficient information. |
| Incomplete outcome data (attrition bias) | High risk | Comment: Sufficient reporting of attrition with reasons, but high attrition (IG: 62%; CG: 53%) might influence outcome. |
| Selective reporting (reporting bias) | Unclear risk | Comment: Insufficient information; No indication to study protocol. |
| Other bias | High risk | Quote: "recruited by convenience sampling" |

| **Fil-Balkan 2018** |  |  |
| --- | --- | --- |
| **Bias** | **Authors' judgement** | **Support for judgement** |
| Random sequence generation (selection bias) | Low risk | Quote: "computerized random number table" |
| Allocation concealment (selection bias) | Unclear risk | Comment: Insufficient information. |
| Blinding of participants and personnel (performance bias) | Unclear risk | Comment: Insufficient information. |
| Blinding of outcome assessment (detection bias) | Unclear risk | Comment: Insufficient information. |
| Incomplete outcome data (attrition bias) | Low risk | Comment: Sufficient reporting and balanced between groups, but high number (IG: 53%; 40%). |
| Selective reporting (reporting bias) | Unclear risk | Comment: Insufficient information; No indication to study protocol. |
| Other bias | High risk | Comment: Measurements in "on-period". |

| **Gandolfi 2017** |  |  |
| --- | --- | --- |
| **Bias** | **Authors' judgement** | **Support for judgement** |
| Random sequence generation (selection bias) | Low risk | Quote: "Computer-generated random number tables (allocation ratio 1 : 1)" |
| Allocation concealment (selection bias) | Unclear risk | Comment: Insufficient information. |
| Blinding of participants and personnel (performance bias) | Unclear risk | Comment: Insufficient information. |
| Blinding of outcome assessment (detection bias) | Low risk | Quote: "Outcomes were assessed by a single examiner blinded to treatment assignment" |
| Incomplete outcome data (attrition bias) | Unclear risk | Comment: Loss to follow up less in both groups (IG: 5%; CG: 11%), but reasons insufficient stated. |
| Selective reporting (reporting bias) | Unclear risk | Comment: Insufficient information; No indication to study protocol. |
| Other bias | High risk | Quote: "Exercise only when in the ON state" |

| **Geraghty 2017** |  |  |
| --- | --- | --- |
| **Bias** | **Authors' judgement** | **Support for judgement** |
| Random sequence generation (selection bias) | Low risk | Quote: "The randomization allocation process was automated and occurred online". "generated by the Internet intervention software". |
| Allocation concealment (selection bias) | Low risk | Quote: "The randomization sequence… was concealed from the trial team". |
| Blinding of participants and personnel (performance bias) | Unclear risk | Comment: Insufficient information. |
| Blinding of outcome assessment (detection bias) | Low risk | Quote: "An independent research assistant who collected outcome data remained blinded to allocation". |
| Incomplete outcome data (attrition bias) | High risk | Comment: Sufficient reporting of attrition with reasons, but not balanced between groups (IG: 30%; CG: 13%). |
| Selective reporting (reporting bias) | High risk | Comment: Indication to trial protocol, but EQ-5D and CEQ outcome reports are missing. |
| Other bias | Unclear risk | - |

| **Hansson 2008** |  |  |
| --- | --- | --- |
| **Bias** | **Authors' judgement** | **Support for judgement** |
| Random sequence generation (selection bias) | High risk | Quote: "Without randomization" |
| Allocation concealment (selection bias) | High risk | Comment: Insufficient information, but different recruitment areas. |
| Blinding of participants and personnel (performance bias) | Unclear risk | Comment: Insufficient information. |
| Blinding of outcome assessment (detection bias) | Low risk | Quote: "The physiotherapist who performed baseline measures and follow-ups was aware of which group the patient belonged to at baseline. At the follow-ups, information about which group the patient belonged to was not available to her." |
| Incomplete outcome data (attrition bias) | High risk | Comment: Sufficient reporting of attrition with reasons but not balanced between groups (IG: 29%; CG: 15%). |
| Selective reporting (reporting bias) | Unclear risk | Comment: Insufficient information; No indication to study protocol. |
| Other bias | Unclear risk | - |

| **Hansson 2015** |  |  |
| --- | --- | --- |
| **Bias** | **Authors' judgement** | **Support for judgement** |
| Random sequence generation (selection bias) | Low risk | Quote: "Using a computer-created random number list" |
| Allocation concealment (selection bias) | Low risk | Quote: "Sealed envelopes" |
| Blinding of participants and personnel (performance bias) | Unclear risk | Quote: "Intervention was performed by another independent physiotherapist"  Comment: Participants insufficient reported. |
| Blinding of outcome assessment (detection bias) | Low risk | Quote: "The physiotherapist who performed the measurements was not aware of which group the patient was included in" |
| Incomplete outcome data (attrition bias) | High risk | Comment: Sufficient reporting of attrition with reasons but not balanced between groups (IG: 34,1%; CG: 6,8%). |
| Selective reporting (reporting bias) | High risk | Comment: Indication to study protocol, but static balance measurements are missing. |
| Other bias | Unclear risk | - |

| **Kyrdalen 2014** |  |  |
| --- | --- | --- |
| **Bias** | **Authors' judgement** | **Support for judgement** |
| Random sequence generation (selection bias) | Low risk | Quote: "A Web-based block randomization procedure with varying group size was used" |
| Allocation concealment (selection bias) | Low risk | Quote: "randomly allocated by a physiotherapist not involved in treatment or assessment during the study". |
| Blinding of participants and personnel (performance bias) | Unclear risk | Comment: single blind RCT, but only outcome assessor is defined as blinded. Insufficient information. |
| Blinding of outcome assessment (detection bias) | Low risk | Quote: "Assessors were blinded to participants’ group assignment" |
| Incomplete outcome data (attrition bias) | Low risk | Comment: Sufficient reporting and balanced between groups (IG: 27,4%; CG: 25,3%). |
| Selective reporting (reporting bias) | Low risk | Comment: Indication to study protocol and all pre-defined outcomes measured. |
| Other bias | Unclear risk | - |

| **Liao 2015** |  |  |
| --- | --- | --- |
| **Bias** | **Authors' judgement** | **Support for judgement** |
| Random sequence generation (selection bias) | Low risk | Quote: "An individual independent of the study selected one of a set of sealed envelopes to assign participants to the VRWii group, TE group, or control group prior to the intervention" |
| Allocation concealment (selection bias) | Low risk | Quote: "sealed envelopes" |
| Blinding of participants and personnel (performance bias) | Unclear risk | Comment: Insufficient information about participants, personnel "training therapist was not blinded". |
| Blinding of outcome assessment (detection bias) | Low risk | Quote: "All outcomes… by a physical therapist blinded to the group assignment" |
| Incomplete outcome data (attrition bias) | Low risk | Comment: Sufficient reporting and balanced between groups. |
| Selective reporting (reporting bias) | Unclear risk | Comment: Insufficient information; No indication to study protocol. |
| Other bias | High risk | Quote: "The measurement and intervention were conducted with the patients in the “on” state"; |

| **Maciaszek 2012** |  |  |
| --- | --- | --- |
| **Bias** | **Authors' judgement** | **Support for judgement** |
| Random sequence generation (selection bias) | Unclear risk | Comment: Insufficient information; only "randomly assigned". |
| Allocation concealment (selection bias) | Unclear risk | Comment: Insufficient information. |
| Blinding of participants and personnel (performance bias) | Unclear risk | Comment: Insufficient information. |
| Blinding of outcome assessment (detection bias) | Unclear risk | Comment: Insufficient information. |
| Incomplete outcome data (attrition bias) | Unclear risk | Comment: Insufficient information. |
| Selective reporting (reporting bias) | Unclear risk | Comment: Insufficient information; No indication to study protocol. |
| Other bias | Unclear risk | - |

| **Reid 2014** |  |  |
| --- | --- | --- |
| **Bias** | **Authors' judgement** | **Support for judgement** |
| Random sequence generation (selection bias) | Low risk | Quote: "randomly allocated"; "An independent statistician generated a randomization sequence" |
| Allocation concealment (selection bias) | Low risk | Quote: "which was placed in sequentially numbered, opaque, sealed envelopes. Participants were blinded as to whether they received a placebo or active intervention. |
| Blinding of participants and personnel (performance bias) | Unclear risk | Quote: "Blinding of participants, but personnel was not blind to group allocation, the therapist attempted to provide the same amount of attention to all participants" |
| Blinding of outcome assessment (detection bias) | Low risk | Quote: "blinded outcome assessment" |
| Incomplete outcome data (attrition bias) | Low risk | Comment: Sufficient reporting and balanced between groups (IG1: 10%; IG2: 7%; CG: 4%). |
| Selective reporting (reporting bias) | High risk | Comment: Indication to study protocol and most pre-specified outcomes reported, but posturography, CROM and sense of motion missing reporting. |
| Other bias | Unclear risk | - |

| **Ribeiro 2017** |  |  |
| --- | --- | --- |
| **Bias** | **Authors' judgement** | **Support for judgement** |
| Random sequence generation (selection bias) | Low risk | Quote: "The randomization program was computer generated using a basic random number generator in blocks" |
| Allocation concealment (selection bias) | Low risk | Quote: "The allocation sequence was concealed by an independent researcher. Group allocation was concealed from the assessors until the end of the study. Sample randomization and allocation were performed by a researcher who was not directly involved in the assessment or intervention of patients." |
| Blinding of participants and personnel (performance bias) | Unclear risk | Quote: "it was not possible to blind neither patients nor therapists involved" |
| Blinding of outcome assessment (detection bias) | Low risk | Quote: "Two assessors blind to the subject’s group assignment evaluated all participants at baseline and after one, five, nine and thirteen weeks, and performed the CRM as needed" |
| Incomplete outcome data (attrition bias) | Low risk | Comment: Sufficient reporting and balanced between groups (IG: 13%; CG: 13%). |
| Selective reporting (reporting bias) | Low risk | Quote: "This clinical trial is recorded in the Brazilian Clinical Trials (RBR-7jkbyg)". Primary outcome not clearly stated (3 primary outcomes described) |
| Other bias | Unclear risk | Quote :"For those interested in participating… to determine their eligibility" |

| **Ricci 2016** |  |  |
| --- | --- | --- |
| **Bias** | **Authors' judgement** | **Support for judgement** |
| Random sequence generation (selection bias) | Low risk | Quote: "randomly assigned"… "block randomization using SPSS software" |
| Allocation concealment (selection bias) | Low risk | Quote: "A researcher not involved in data collection generated randomization." |
| Blinding of participants and personnel (performance bias) | Unclear risk | Quote: Participants: "they were blinded in relation to the protocol they were involved"; Personnel: "treating therapist was informed… just before their first session" |
| Blinding of outcome assessment (detection bias) | Low risk | Quote :"..only the outcome assessor was unaware of the treatment allocation" |
| Incomplete outcome data (attrition bias) | Unclear risk | Comment: Insufficient reporting divided in groups. |
| Selective reporting (reporting bias) | High risk | Comment: Indication to study protocol, but MMSE and GDS missing. |
| Other bias | Unclear risk | - |

| **Rossi-Izquierdo 2018** |  |  |
| --- | --- | --- |
| **Bias** | **Authors' judgement** | **Support for judgement** |
| Random sequence generation (selection bias) | Low risk | Quote: "An n = 20 block balanced randomization sequence was used." |
| Allocation concealment (selection bias) | Unclear risk | Comment: Insufficient reporting. |
| Blinding of participants and personnel (performance bias) | Unclear risk | Comment: Insufficient reporting. |
| Blinding of outcome assessment (detection bias) | Unclear risk | Comment: Insufficient reporting. |
| Incomplete outcome data (attrition bias) | Low risk | Comment: Sufficient reporting and quite balanced between groups (IG1: =20%; IG2: 11,7%; IG3: 31,4%; CG: 20%). |
| Selective reporting (reporting bias) | Low risk | Comment: Indication to study protocol and all pre-specified oucomes reported. |
| Other bias | Unclear risk | - |

| **Smaerup 2016** |  |  |
| --- | --- | --- |
| **Bias** | **Authors' judgement** | **Support for judgement** |
| Random sequence generation (selection bias) | Low risk | Quote: "computer" |
| Allocation concealment (selection bias) | Unclear risk | Comment: Insufficient reporting. |
| Blinding of participants and personnel (performance bias) | Unclear risk | Comment: Insufficient reporting. |
| Blinding of outcome assessment (detection bias) | Low risk | Quote: "Outcome assessor blinded" |
| Incomplete outcome data (attrition bias) | Low risk | Comment: Sufficient reported and balanced between groups (IG: 9%; CG: 6%) |
| Selective reporting (reporting bias) | Low risk | Comment: Indication to study protocol and all pre-specified outcomes reported. |
| Other bias | Unclear risk | - |

| **Stam 2018** |  |  |
| --- | --- | --- |
| **Bias** | **Authors' judgement** | **Support for judgement** |
| Random sequence generation (selection bias) | Low risk | Quote: "Practices were stratified by list size into three strata: practices with up to 400, 400 to 800, and over 800 patients of 65 years and older. For each stratum, block randomisation with varying block size was used to create similar distributions in both study arms. The investigator was blinded to the size of each block" |
| Allocation concealment (selection bias) | Low risk | Quote: "Practices were randomised by a researcher who was blinded to their identity (concealment of allocation) before the inclusion of patients began" |
| Blinding of participants and personnel (performance bias) | Unclear risk | Quote: "Blinding of patients and health care professionals was not possible due to the nature of the interventions" |
| Blinding of outcome assessment (detection bias) | Unclear risk | Quote: "Blinding of outcome assessors was therefore not possible" |
| Incomplete outcome data (attrition bias) | Low risk | Comment: Sufficient reported and balanced between groups (IG: 19,2%; CG: 21%). |
| Selective reporting (reporting bias) | Low risk | Comment: Indication to study protocol and all pre-specified outcomes reported. |
| Other bias | Unclear risk | - |

| **Yang 2012** |  |  |
| --- | --- | --- |
| **Bias** | **Authors' judgement** | **Support for judgement** |
| Random sequence generation (selection bias) | Low risk | Quote: "computer-generated random numbers" |
| Allocation concealment (selection bias) | Unclear risk | Quote: "list was managed by a researcher who was not involved in recruiting or assessing participants. Group assignment was made for each participant after the baseline assessment." |
| Blinding of participants and personnel (performance bias) | Unclear risk | Comment: Insufficient information. |
| Blinding of outcome assessment (detection bias) | Low risk | Quote: "This study was single-blinded, as only the assessors were blinded to group assignment." |
| Incomplete outcome data (attrition bias) | High risk | Comment: Sufficient reported and quite balanced between groups (IG: 28%; CG: 25%) |
| Selective reporting (reporting bias) | Low risk | Comment: Indication to study protocol and all pre-specified outcomes reported. |
| Other bias | High risk | Comment: Recruitment method implicates more volunteers |

| **Yen 2011** |  |  |
| --- | --- | --- |
| **Bias** | **Authors' judgement** | **Support for judgement** |
| Random sequence generation (selection bias) | Low risk | Quote: "block randomized design" |
| Allocation concealment (selection bias) | Unclear risk | Comment: Insufficient information. |
| Blinding of participants and personnel (performance bias) | Unclear risk | Comment: Patients: Insufficient information; Personnel: "blinded to group assignment" |
| Blinding of outcome assessment (detection bias) | Low risk | Quote: "administered by one blinded, trained physical therapist" |
| Incomplete outcome data (attrition bias) | Low risk | Comment: Sufficient reported and quite balanced between groups (IGs: 14%; CG 36%) |
| Selective reporting (reporting bias) | Low risk | Comment: Indication to study protocol and all pre-specified outcomes reported. |
| Other bias | High risk | Quote: "in on phase" |

| **Zambare 2015** |  |  |
| --- | --- | --- |
| **Bias** | **Authors' judgement** | **Support for judgement** |
| Random sequence generation (selection bias) | High risk | Comment: Non-RCT |
| Allocation concealment (selection bias) | Unclear risk | Comment: Insufficient information. |
| Blinding of participants and personnel (performance bias) | Unclear risk | Comment: Insufficient information. |
| Blinding of outcome assessment (detection bias) | Unclear risk | Comment: Insufficient information. |
| Incomplete outcome data (attrition bias) | Low risk | Comment: Sufficient reported and balanced between groups (Both groups 0%) |
| Selective reporting (reporting bias) | Unclear risk | Comment: Insufficient information. |
| Other bias | Unclear risk | - |
