# Supplementary material for: Physical therapy interventions for older people with vertigo, dizziness and balance disorders addressing mobility and participation: a systematic review
Source: BMC Geriatr. 2020 Nov 23;20:494. doi: 10.1186/s12877-020-01899-9 (PMC7684969; doi:10.1186/s12877-020-01899-9)
Supplement: Supplementary file 4 — Additional file 4. Primary and secondary outcome measures. [file 12877_2020_1899_MOESM4_ESM.docx]

**Additional file 4** Primary and secondary outcome measures

| **Primary outcome measures** | | | | | | | | | | | | | | | | | | | | | | | | | | | | | |  |  |
| --- | --- | --- | --- | --- | --- | --- | --- | --- | --- | --- | --- | --- | --- | --- | --- | --- | --- | --- | --- | --- | --- | --- | --- | --- | --- | --- | --- | --- | --- | --- | --- |
| **Body structure and functions** | | | | | | **Activities and participation** | | | | | | | | | | | | | | | | | | | | | | | |  |  |
| ***Postural control*** | | | | | | ***Balance*** | | | | | | | | | | | | | | | | | | | |  |  |  |  |  |  |
| LOS | | | [32, 41, 44] | | | BBS | | | | | | | | | [34, 48] | | | | | | | | | |  |  |  |  |  |  |  |
| mCTSIB | | | [41, 44] | | | DGI | | | | | | | | | [41, 42] | | | | | | | | | |  |  |  |  |  |  |  |
| tandem standing with eyes open and closed | | | [37] | | | One leg stand test | | | | | | | | | [43] | | | | | | | | | |  |  |  |  |  |  |  |
| RWS | | | [44] | | | Walking in a modified figure of eight | | | | | | | | | [37] | | | | | | | | | |  |  |  |  |  |  |  |
| SOT | | | [32] | | |  | | | | | | | | |  | | | | | | | | | |  |  |  |  |  |  |  |
|  | | |  | | |  | | | | | | | | | |  | | | | | | | | | |  |  |  |  |  |  |
| ***VDB symptoms*** | | | | | | ***Impact of VDB on ADL*** | | | | | | | | | | | | | | | | | | | |  |  |  |  |  |  |
| VSS-SF | | | [35] | | | DHI | | | | | | | | | [49] | | | | | | | | | |  |  |  |  |  |  |  |
| VAS dizziness intensity | | | [40] | | | | | | | | |  | | | | | | |  | | | | | | | | | | |  |  |
| **Secondary outcome measures** | | | | | | | | | | | | | | | | | | | | | | | | | | | | | | |  |
| **Body structure and functions** | | | | | | | **Activities and participation** | | | | | | | | | | | | | **Quality of life** | | | | **Falls** | | | | | |  |  |
| ***Postural control*** | | | | | | ***Balance*** | | | | | | | | | | | | | | | PDQ-39 | | [30, 34] | FES-I | | | [48, 51] | |  |  |  |
| SOT | | | [33, 38, 45, 51] | | | BBS | | | | | | | [30, 46, 47, 50] | | | | | | | | SF-36 | | [48] | Risk of falls | | | [36] | |  |  |  |
| LOS | | | [39, 41, 51] | | | DGI | | | | | | | [30, 34, 43, 50] | | | | | | | | QoL | | [49] | Fall rate | | | [42] | |  |  |  |
| mCTSIB | | | [30, 41] | | | ABC | | | | | | | [30, 34] | | | | | | | | AQol | | [44] | Fall frequency | | | [49] | |  |  |  |
| SOLEO / SOLEC | | | [36, 37] | | | FGA | | | | | | | [38] | | | | | | | | SF-12 | | [43] | MFES | | | [44] | |  |  |  |
| CDP-SOT | | | [47] | | |  | | | | | | |  | | | | | | | |  | |  | Likelihood of falls | | | [50] | |  |  |  |
| CDP-Sensory | | | [47] | | | ***Mobility*** | | | | | | |  | | | | | | | |  | |  | Falls | | | [34] | |  |  |  |
| Tandem standing with eyes open and closed | | | [36] | | | TUG | | | | | | | [30, 32, 42, 46, 47, 48, 51] | | | | | | | |  | |  |  | | |  | |  |  |  |
| Postural sway | | | [37] | | | FRT | | | | | | | [44, 47] | | | | | | | | |  | |  | | | |  | | |  |
| Vibration sense | | | [37] | | | Walking heel to toe on a line | | | | | | | [36, 37] | | | | | | | |  | | |  | | | |  | | |  |
| Head shake test | | | [37] | | | Multidirectional FR | | | | | | | [42] | | | |  | | | | | | | | | | | | | | |
| US sway | | | [41] | | | Steps in figure of eight | | | | | | | [36] | | | |  |  |  |  |  |  |  |  |  |  |  |  |  |  |  |
| Tandem end sway | | | [41] | | | Walking as fast as possible | | | | | | | [37] | | | |  |  |  |  |  |  |  |  |  |  |  |  |  |  |  |
| Romberg | | | [42] | | | Walk across speed | | | | | | | [41] | | | | |  |  |  |  |  |  |  |  |  |  |  |  |  |  |
| Tandem stand | | | [42] | | | Walk across test | | | | | | | [44] | | | |  |  |  |  |  |  |  |  |  |  |  |  |  |  |  |
| Sensorial | | | [42] | | | 10-MW | | | | | | | [34] | | | |  |  |  |  |  |  |  |  |  |  |  |  |  |  |  |
| Unipedal | | | [42] | | | 8-foot up and go test | | | | | | | [39] | | | |  |  |  |  |  |  |  |  |  |  |  |  |  |  |  |
| Static balance (stabilometry) | | | [46] | | | HAP-AAS | | | | | | | [44] | | | |  |  |  |  |  |  |  |  |  |  |  |  |  |  |  |
|  | | |  | | | SQT | | | | | | | [44] | | | |  |  |  |  |  |  |  |  |  |  |  |  |  |  |  |
|  | | |  | | |  | | | | | | |  | | | |  |  |  |  |  |  |  |  |  |  |  |  |  |  |  |
|  | | |  | | |  | | | | | | |  | | | |  |  |  |  |  |  |  |  |  |  |  |  |  |  |  |
|  | | |  | | |  | | | | | | |  | | | |  |  |  |  |  |  |  |  |  |  |  |  |  |  |  |
| ***Strength*** | | | | | | ***Impact of VDB on ADL*** | | | | | | | |  |  |  |  |  |  |  |  |  |  |  |  |  |  |  |  |  |  |
| 5x-STS | | | [37, 44, 48] | | | DHI | | | | | | | [31, 35, 36, 40, 41, 43, 51] | | | | | | | | | | | | | | | | |  |  |
| STS | | | [42, 44] | | |  | | | |  | | | |  |  |  |  |  |  |  |  |  |  |  |  |  |  |  |  |  |  |
| Muscle strength of lower extremity | | | [33, 38, 44] | | | ***Functional status*** | | | |  | | | |  |  |  |  |  |  |  |  |  |  |  |  |  |  |  |  |  |  |
| Handgrip strength | | | [42] | | | UPDRS | | | | | | | [30, 47] | | | |  |  |  |  |  |  |  |  |  |  |  |  |  |  |  |
| Chair stand test | | | [43] | | |  | | | | | | |  | | | |  |  |  |  |  |  |  |  |  |  |  |  |  |  |  |
|  | | |  | | | ***Other*** | | | | | | |  | | | |  |  |  |  |  |  |  |  |  |  |  |  |  |  |  |
|  | | |  | | | VRT | | | | | | | [45] | | | |  |  |  |  |  |  |  |  |  |  |  |  |  |  |  |
| ***Gait parameters*** | | | | | | FIM | | | | | | | [46] | | | |  |  |  |  |  |  |  |  |  |  |  |  |  |  |  |
| Walking speed | | | [44] | | |  |  |  |  |  |  |  |  |  |  |  |  |  |  |  |  |  |  |  |  |  |  |  |  |  |  |
| Gait velocity | | | [38] | | |  |  |  |  |  |  |  |  |  |  |  |  |  |  |  |  |  |  |  |  |  |  |  |  |  |  |
| Stride length | | | [38] | | |  |  |  |  |  |  |  |  |  |  |  |  |  |  |  |  |  |  |  |  |  |  |  |  |  |  |
|  | | |  | | |  |  |  |  |  |  |  |  |  |  |  |  |  |  |  |  |  |  |  |  |  |  |  |  |  |  |
| ***VDB symptoms*** | | | | | |  |  |  |  |  |  |  |  |  |  |  |  |  |  |  |  |  |  |  |  |  |  |  |  |  |  |
| VAS dizziness intensity | | | [40, 41, 43] | | |  |  |  |  |  |  |  |  |  |  |  |  |  |  |  |  |  |  |  |  |  |  |  |  |  |  |
| VAS dizziness frequency | | | [40, 49] | | |  |  |  |  |  |  |  |  |  |  |  |  |  |  |  |  |  |  |  |  |  |  |  |  |  |  |
|  | | |  | | |  |  |  |  |  |  |  |  |  |  |  |  |  |  |  |  |  |  |  |  |  |  |  |  |  |  |
|  |  |  | |  | | | |  |  |  |  |  |  |  |  |  |  |  |  |  |  |  |  |  |  |  |  |  |  |  |  |
| ***Psychological symptoms*** | |  | |  | | | |  |  |  |  |  |  |  |  |  |  |  |  |  |  |  |  |  |  |  |  |  |  |  |  |
| HADS | | | [35] | |  | | | |  |  |  |  |  |  |  |  |  |  |  |  |  |  |  |  |  |  |  |  |  |  |  |
| GAD-7 | | | [49] | |  |  |  |  |  |  |  |  |  |  |  |  |  |  |  |  |  |  |  |  |  |  |  |  |  |  |  |
| PHQ-PD, PHQ-9 | | | [49] | |  |  |  |  |  |  |  |  |  |  |  |  |  |  |  |  |  |  |  |  |  |  |  |  |  |  |  |
| VAS-FOF | | | [50] | |  |  |  |  |  |  |  |  |  |  |  |  |  |  |  |  |  |  |  |  |  |  |  |  |  |  |  |
|  | | |  | |  |  |  |  |  |  |  |  |  |  |  |  |  |  |  |  |  |  |  |  |  |  |  |  |  |  |  |
| ***Health status*** | | | | |  |  |  |  |  | |  |  |  |  |  |  |  |  |  |  |  |  |  |  |  |  |  |  |  |  |  |
| EQ5D-VAS | | | [37] | |  |  |  |  |  |  |  |  |  |  |  |  |  |  |  |  |  |  |  |  |  |  |  |  |  |  |  |
|  | | |  | |  |  |  |  |  |  |  |  |  |  |  |  |  |  |  |  |  |  |  |  |  |  |  |  |  |  |  |
| ***Endurance*** | | |  | |  |  |  |  |  |  |  |  |  |  |  |  |  |  |  |  |  |  |  |  |  |  |  |  |  |  |  |
| Step test | | | [44] | |  |  |  |  |  |  |  |  |  |  |  |  |  |  |  |  |  |  |  |  |  |  |  |  |  |  |  |
|  | | |  | |  |  |  |  |  |  |  |  |  |  |  |  |  |  |  |  |  |  |  |  |  |  |  |  |  |  |  |
| ***Other*** | | |  | |  |  |  |  |  |  |  |  |  |  |  |  |  |  |  |  |  |  |  |  |  |  |  |  |  |  |  |
| VAS pain intensity | | | [40] | |  |  |  |  |  |  |  |  |  |  |  |  |  |  |  |  |  |  |  |  |  |  |  |  |  |  |  |
| Body symmetry (barometry) | | | [46] | |  |  |  |  |  |  |  |  |  |  |  |  |  |  |  |  |  |  |  |  |  |  |  |  |  |  |  |
| Motions sensitivity | | | [43] | |  |  |  |  |  |  |  |  |  |  |  |  |  |  |  |  |  |  |  |  |  |  |  |  |  |  |  |
| passive knee joint repositioning test | | | [33] | |  | | | |  |  |  |  |  |  |  |  |  |  |  |  |  |  |  |  |  |  |  |  |  |  |  |
|  | | |  | | |  | | | | | | |  | | | |  |  |  |  |  |  |  |  |  |  |  |  |  |  |  |
| 10-MW = Ten meter walking; 5x-STS = Five times sit to stand test; ABC = Activities-specific balance confidence; AQoL = Assessment of quality of life; BBS = Berg balance scale; CDP-Sensory = Computerized dynamic posturography – sensory analysis; CDP-SOT = Computerized dynamic posturography – Sensory organization test; CG = Control group; CI = Confidence interval; CoG = Comparison group; DGI = Dynamic gait index; DHI = Dizziness handicap inventory; EQ5D-VAS = Visual analogue scale of five dimensions EuroQol; FES-I = 7 item falls efficacy scale international; FGA = Functional gait assessment; FIM = 7-level functional independence measure; FRIDs = Fall-risk-increasing drugs; FRT = Functional reach test; GAD-7 = generalised anxiety disorder assessment 7 subscale; HADS = Hospital anxiety and depression scale; HAP-AAS = Human activity profile–adjusted activity score; IG = Intervention group; LOS = Limits of stability; mCTSIB = modified clinical test of sensory interaction on balance; MFES = Modified falls efficacy scale; Multidirectional FR = Multidirectional functional reach; MVL = Movement velocity; MXE = Maximum excursion; PDQ-39 = Parkinson's Disease Questionnaire; PHQ-PD = Patient Health Questionnaire Panic Module; PHQ-9 = patient health questionnaire-9; PREF = Visual preference score; QoL = Quality of life; RWS = Rhythmic weight shift; SF-12 = 12 item short form assessment of quality of life; SF-36 MH = 36 item short form assessment health-related quality of life mental health; SF-36 PH = 36 item short form assessment health-related quality of life physical health; SNAGs = Sustained natural apophyseal glides; SOLEC = Standing on one leg with eyes closed; SOLEO = standing on one leg with eyes open; SOM = Somatosensory system score; SOT = Sensory organization test; SQT = Step quick turn test; STS = Sit to stand; TUG = Timed up and go test; UPDRS = Unified Parkinson´s disease rating scale; US = Unilateral stance test; VAS = Visual analogue scale; VAS-FOF = Visual analogue scale for fear of fall; VEST = Vestibular system score; VIS = Visual system score; VRT = verbal reaction time; vs. = versus; VSS-SF = Short form of vertigo symptom scale; WA = walk across test. | | | | | | | | | | | | | | | | | | | | | | | | | | | | | |  |  |
