# Supplementary material for: Physical therapy interventions for older people with vertigo, dizziness and balance disorders addressing mobility and participation: a systematic review
Source: BMC Geriatr. 2020 Nov 23;20:494. doi: 10.1186/s12877-020-01899-9 (PMC7684969; doi:10.1186/s12877-020-01899-9)
Supplement: Supplementary file 5 — Additional file 5. Summary of study results. [file 12877_2020_1899_MOESM5_ESM.docx]

**Additional file 5** Summary of study results

| **Reference** | **Change direction** | **Body structure and function** | **Change direction** | **Activities and participation** | **Change direction** | **Quality of life** | **Change direction** | **Falls** | **Change direction** | **Further outcomes** |
| --- | --- | --- | --- | --- | --- | --- | --- | --- | --- | --- |
| **Canal repositioning manoeuvre** | | | | | | | | | | |
| André [31] |  | - | ↔ | *vs. CoG1:*  Advantage of -5,01 points (p = .009) in physical subscale of DHI between.  No difference in emotional and functional subscales and **general score.** |  | - |  | - |  | - |
|  |  | - | ↔ | *vs. CoG2:*  No difference in general score and subscales of DHI. |  | - |  | - |  | - |
| **Vestibular rehabilitation** | | | | | | | | | | |
| Acarer [30] | ↔ | Advantage of -.07 (p < .05) in mCTSIB with eyes open on firm surface.  Advantage of -.08 (p < .05) in mCTSIB with eyes open on foam surface.  No difference in mCTSIB with eyes closed on firm and foam surface and in **total score.** | Unclear  (3/5) | Advantage of +9 points (p < .05) in BBS.  Advantage of +4 points (p < .05) in DGI.  Advantage of +27,5 points (p < .05) in ABC.  No difference in UPDRS and TUG. | ↔ | No difference in PDQ-39. |  | - |  | - |
| Fil-Balkan [47] | Unclear  (3/7) | Advantage of +24.16 (p = .027) in position 5^th^ and of +12.8 (p = .042) **composite** in CDP-SOT.  No difference in position 6^th^ of CDP-SOT.  Advantage of +25.43 (p = .048) in VEST of  No difference in SOM, VIS and PREF in CDP-Sensory. | Unclear  (2/4) | No difference in UPDRS.  Advantage of +10.34 points (p = .037) in BBS.  Advantage of -4.11 seconds (p = .002) in TUG.  No difference in FRT. |  | - |  | - |  | - |
| Geraghty [35] | ↑ | Advantage of - 2.38 points (p = .03) univariate and -2.26 points (p = .02) multivariate in **VSS-SF total score.**  No difference in VSS-SF vertigo subscale score.  Advantage of -1.38 (p < .001) univariate and -1.33 (p < .001) multivariate of VSS-SF autonomic symptoms subscale score.  No difference in HADS scores. | ↑ | Advantage of -5.58 points (p= .01) univariate and multivariate in DHI. |  | - |  | - | ↑ | Advantage of patient-reported improvement (IG 64.2% and CG 41.0% of patients)  0.38 (0.25 to 0.59; p <.001) 0.41 (0.26 to 0.65; p <.001)  18 non–dizziness related hospitalizations at the end of the trial (IG: 10; CG: 8). |
| Hansson [36] | Unclear  (1/4) | Advantage of +1 second (p = .038) in SOLEC.  No difference in SOLEO, tandem standing with eyes open and closed. | Unclear  (1/3) | No difference in **DHI total** score and subscales.  Advantage of -2 steps (p = .044) in walking heel to toe.  No difference in steps in figure of eight. |  | - | ↔ | No difference in risk of falls: 40 falls occured (31 intrinsic; 26 caused by vertigo; 9 extrinsic) with no difference between groups. Poor ability to stand in tandem stance doubled the risk for falls. |  | - |
| Hansson [37] | ↔ | No difference in **tandem standing with eyes open and closed,** SOLEO, SOLEC, 5x-STS test, postural sway, vibration sense, head shake test and EQ5D-VAS. | ↔ | No difference in **walking in a modified figure of eight**, walking heel to toe on a line, walking as fast as possible for 30 m with one turn after 15 m. |  | - |  | - |  | - |
| Kyrdalen [48] | ↑ | Advantage of +2.2 seconds (p = .005) in 5x-STS. | ↔ | Advantage in -2.4 seconds (p = .038) of TUG.  No difference in **BBS**. | ↔ | No difference in SF-36 PH and MH. | ↔ | No difference in FES-I. |  | - |
| Ribeiro [41] | Unclear  (2/11) | No difference in **mCTSIB** scores, US sway scores, **MVL (LOS)** and VAS dizziness intensity.  Advantage of + 17 % in **MXE (LOS)** and of -1 seconds in tandem end sway (p < .05). | ↑ | Advantage of +4 points (p = .05) in **DGI**.  No difference in WA speed and DHI total and subscales. |  | - |  | - |  | - |
| Ricci [42] | ↔ | No difference in STS, Romberg, tandem stand, sensorial, unipedal, handgrip strength. | ↔ | No difference in **DGI**, TUG total score, TUG cognitive, TUG manual, multidirectional FR. |  | - | ↔ | No difference in fall rate. |  | - |
| Rossi-Izquierdo [51] | ↔ | *vs. CG:*  No difference in SOT and LOS. | ↔ | *vs. CG:*  No difference in DHI and TUG. |  | - | ↔ | *vs. CG:*  No difference in FES-I. |  | - |
|  | ↔ | *vs. CoG1:*  No difference in SOT and LOS. | ↔ | *vs. CoG1:*  No difference in DHI and TUG. |  | - | ↔ | *vs. CoG1:*  No difference in FES-I. |  | - |
|  | ↔ | *vs. CoG2:*  No difference in SOT and LOS. | ↔ | *vs. CoG2:*  No difference in DHI and TUG. |  | - | ↔ | *vs. CoG2:*  No difference in FES-I. |  | - |
| Stam [49] | ↔ | No difference in VAS dizziness frequency and presence of anxiety and depressive disorder (GAD-7, PHQ-PD, PHQ-9). | ↔ | No difference in **DHI.** | ↔ | No difference in QoL. | ↔ | No difference in fall frequency (VAS). |  | Advantage (p = .02) in number of FRIDs. |
| Yang [44] | ↔ | Advantage of +2.10 steps/15s (p ≤ .001) in step test (worse leg) and of + .02 kg/kg hip abductor muscle strength (worse side).  No difference in **mCTSIB, LOS, RWS**, STS, 5x-STS, Muscle strength of quadriceps and dorsiflexors and walking speed. | Unclear  (3/4) | Advantage of -2.17 cm in WA (p ≤ .001).  Advantage of +2.95 cm in FRT (p ≤ .001).  Advantage of +4.57 points of HAP-AAS (p ≤ .001).  No difference in SQT. | ↔ | No difference in AQoL. | ↔ | No difference in MFES. |  |  |
| Zambare [50] | ↑ | Advantage of -0.77 points (p = .030) in VAS-FOF. | Unclear  (1/2) | No difference in BBS.  Advantage of +1.3 points (p = .013) in DGI. |  |  | ↔ | No difference in likelihood of falls. |  |  |
| **Computer assisted vestibular rehabilitation** | | | | | | | | | | |
| Barcala [46] | ↔ | No difference in body symmetry (baropodometry), static balance (stabilometry) | ↔ | No difference in BBS, TUG and FIM. |  | - |  | - |  | - |
| Gandolfi [34] |  | - | ↔ | No difference in **BBS**, ABC, 10-MW and DGI. | ↔ | No difference in PDQ-39. | ↔ | No difference in falls. |  | - |
| Liao [38] | Unclear  (9/11) | *vs. CoG:*  Advantage of + 12,87 cm/s (p < .05) in velocity.  Advantage of +15.41 cm (p < .05) in stride length.  Advantage of + 16.5 N in hipflexors, of + 14.6 N in knee flexors, of + 28.1 N in knee extensors of + 37.5 N in ankle dorsiflexors and of + 25.5 N in ankle plantarflexors (p < .05).  No difference in somatosensory ratios of SOT and strength of hip extensors.  Advantage of + 8.8 in visual and + 20.5 (p < .05) in vestibular ratio of SOT. | ↑ | *vs. CoG:*  Advantage of +4.59 points (p < .05) in FGA. |  | - |  | - |  | - |
|  | Unclear  (8/11) | *CG vs. CoG:*  Advantage of + 6.93 cm/s (p < .05) in velocity.  Advantage of +13.2 cm (p < .05) in stride length.  Advantage of + 26.9 N in hipflexors, of + 25.3 N in knee flexors, of + 28.3 N in knee extensors of + 23.9 N in ankle dorsiflexors and of + 19.6 N in ankle plantarflexors (p < .05).  No difference in strength of hip extensors, somatosensory and visual ratios of SOT.  Advantage of + 12.7 (p < .05) in vestibular ratio of SOT. | ↑ | *CG vs. CoG:*  Advantage of +3.09 points (p < .05) in FGA. |  |  |  |  |  |  |
| Smaerup [43] | ↔ | No difference in Motion Sensitivity, VAS vertigo intensity and Chair stand test. | ↔ | No difference in **One leg stand**, DGI and DHI. | ↔ | No difference in SF-12 physical and mental composite. |  | - |  | - |
| Yen [45] | ↔ | No difference in equilibrium scores of SOT and sensory ratios. | ↔ | No difference in VRT. |  | - |  | - |  | - |
| **Tai Chi as vestibular rehabilitation** | | | | | | | | | | |
| Au-Yeung [32] | Unclear  (5/17) | Advantage (p = .005) in reaction time of nonaffected side in **LOS.**  No difference in reaction time of all other sides in **LOS.**  Advantage (p = .005) in end-point excursion of non-affected and affected side and (p = .05) backwards and forwards in **LOS.**  No difference in all conditions of equilibrium score and sensory ratios of **SOT.** | ↔ | No difference in TUG. |  | - |  | - |  | - |
| Chen [33] | Unclear  (3/6) | Advantage of - 30.1 % (p = .032) of absolute angle error of passive knee joint repositioning test.  No difference in concentric isokinetic knee extensor or flexor strength of dominant leg.  Advantage of +59,7 % (p = .006) in visual ratio and of + 50,3 % (p = .048) in vestibular ratio of SOT.  No difference in somatosensory ratio. |  | - |  | - |  | - |  | - |
| Maciaszek [39] | ↑ | Advantage of + .61 cm (p = 0.050) forward,  +1.37 cm (p = .024) backward and + 28.57 cm^2^ (p = .002) in **maximal sway area of LOS.**  No difference in right- and leftward of LOS. | ↑ | Advantage of - .24 s (p = .003) in 8 foot up and go. |  | - |  | - |  | - |
| **Manual therapy** | | | | | | | | | | |
| Reid [40] | ↑ | *vs. CG:*  Advantage of -18.4 (p = .01) in **VAS dizziness intensity.**  Advantage of -0.9 (p < .001) in dizziness frequency.  No difference in VAS pain. | ↔ | *vs. CG*  No difference in DHI. |  | - |  | - |  | Advantage (p < .05) in global perceived effects of patients.  4 reported mild transient pain in their lower cervical spine or upper arm after SNAGs or self-administered  SNAGs. None of the symptoms lasted  longer than 24 hours. |
|  | ↔ | *vs. CoG:*  No difference in **VAS dizziness intensity**, dizziness frequency and VAS pain. | ↓ | *vs. CoG:*  Disadvantage of 7.6 points (p = .04) in DHI. |  | - |  | - |  |  |

**Bold letters**: weighted outcome; **Bold and underlined letters**: stated primary outcome; ↑ = up arrow means significant effect between groups in last follow-up; ↔ = means no difference between groups in last-follow-up; Unclear (3/5) = mixed results (3 positive outcomes out of 5 outcomes); 10-MW = Ten meter walking; 5x-STS = Five times sit to stand test; ABC = Activities-specific balance confidence; AQoL = Assessment of quality of life; BBS = Berg balance scale; CDP-Sensory = Computerized dynamic posturography – sensory analysis; CDP-SOT = Computerized dynamic posturography – Sensory organization test; CG = Control group; CI = Confidence interval; CoG = Comparison group; DGI = Dynamic gait index; DHI = Dizziness handicap inventory; EQ5D-VAS = Visual analogue scale of five dimensions EuroQol; FES-I = 7 item falls efficacy scale international; FGA = Functional gait assessment; FIM = 7-level functional independence measure; FRIDs = Fall-risk-increasing drugs; FRT = Functional reach test; GAD-7 = generalised anxiety disorder assessment 7 subscale; HADS = Hospital anxiety and depression scale; HAP-AAS = Human activity profile–adjusted activity score; IG = Intervention group; LOS = Limits of stability; mCTSIB = modified clinical test of sensory interaction on balance; MFES = Modified falls efficacy scale; Multidirectional FR = Multidirectional functional reach; MVL = Movement velocity; MXE = Maximum excursion; PDQ-39 = Parkinson's Disease Questionnaire; PHQ-PD = Patient Health Questionnaire Panic Module; PHQ-9 = patient health questionnaire-9; PREF = Visual preference score; QoL = Quality of life; RWS = Rhythmic weight shift; SF-12 = 12 item short form assessment of quality of life; SF-36 MH = 36 item short form assessment health-related quality of life mental health; SF-36 PH = 36 item short form assessment health-related quality of life physical health; SNAGs = Sustained natural apophyseal glides; SOLEC = Standing on one leg with eyes closed; SOLEO = standing on one leg with eyes open; SOM = Somatosensory system score; SOT = Sensory organization test; SQT = Step quick turn test; STS = Sit to stand; TUG = Timed up and go test; UPDRS = Unified Parkinson´s disease rating scale; US = Unilateral stance test; VAS = Visual analogue scale; VAS-FOF = Visual analogue scale for fear of fall; VEST = Vestibular system score; VIS = Visual system score; VRT = verbal reaction time; vs. = versus; VSS-SF = Short form of vertigo symptom scale; WA = walk across test.
