# Supplementary material for: Physical therapy interventions for older people with vertigo, dizziness and balance disorders addressing mobility and participation: a systematic review
Source: BMC Geriatr. 2020 Nov 23;20:494. doi: 10.1186/s12877-020-01899-9 (PMC7684969; doi:10.1186/s12877-020-01899-9)
Supplement: Supplementary file 6 — Additional file 6. Harvest plots summarizing effects of interventions. [file 12877_2020_1899_MOESM6_ESM.docx]

**Additional file 6** Harvest plots summarizing effects of interventions


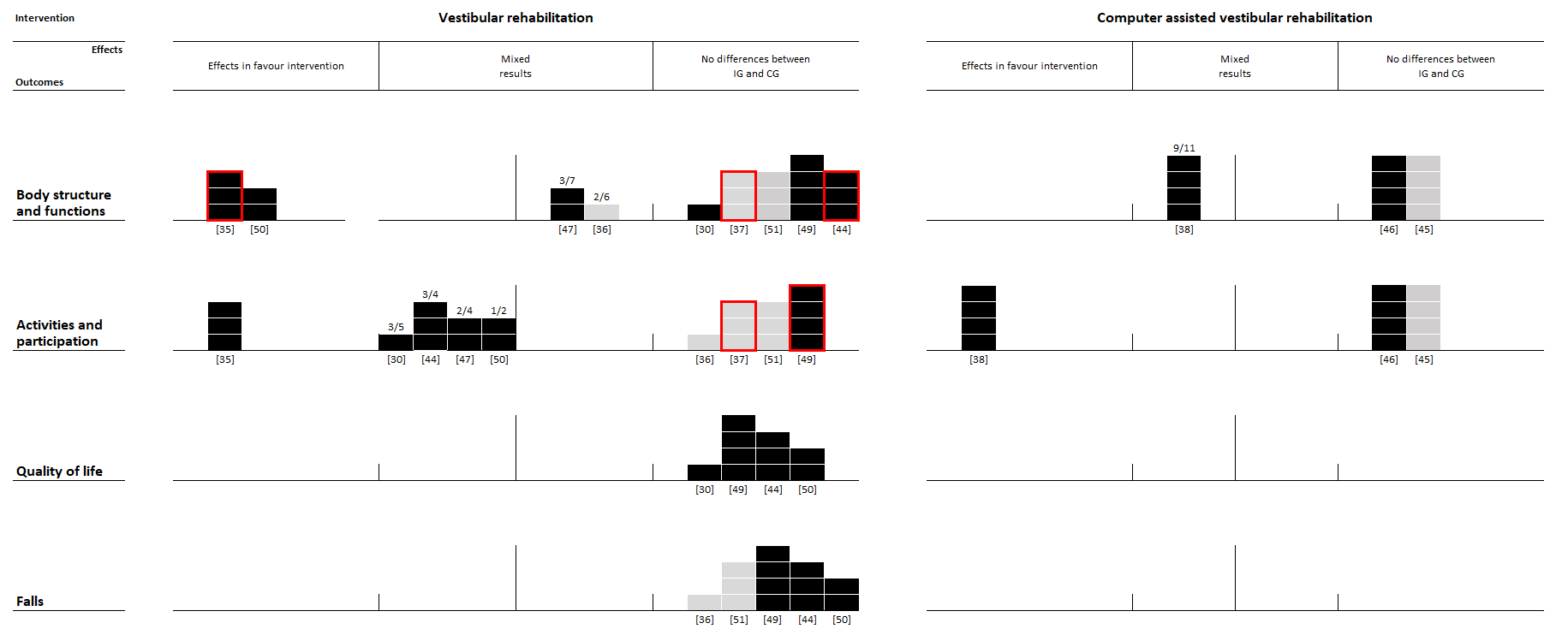


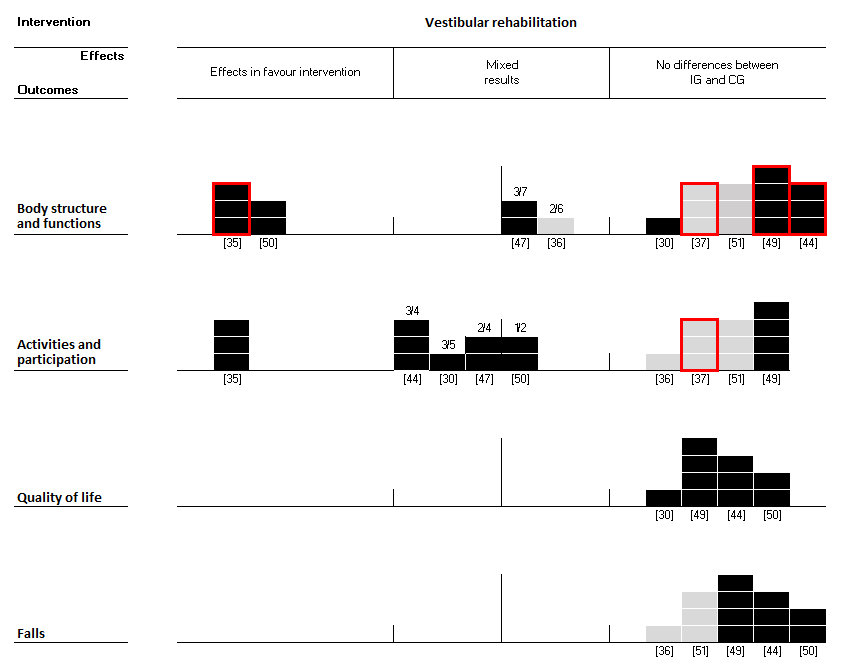

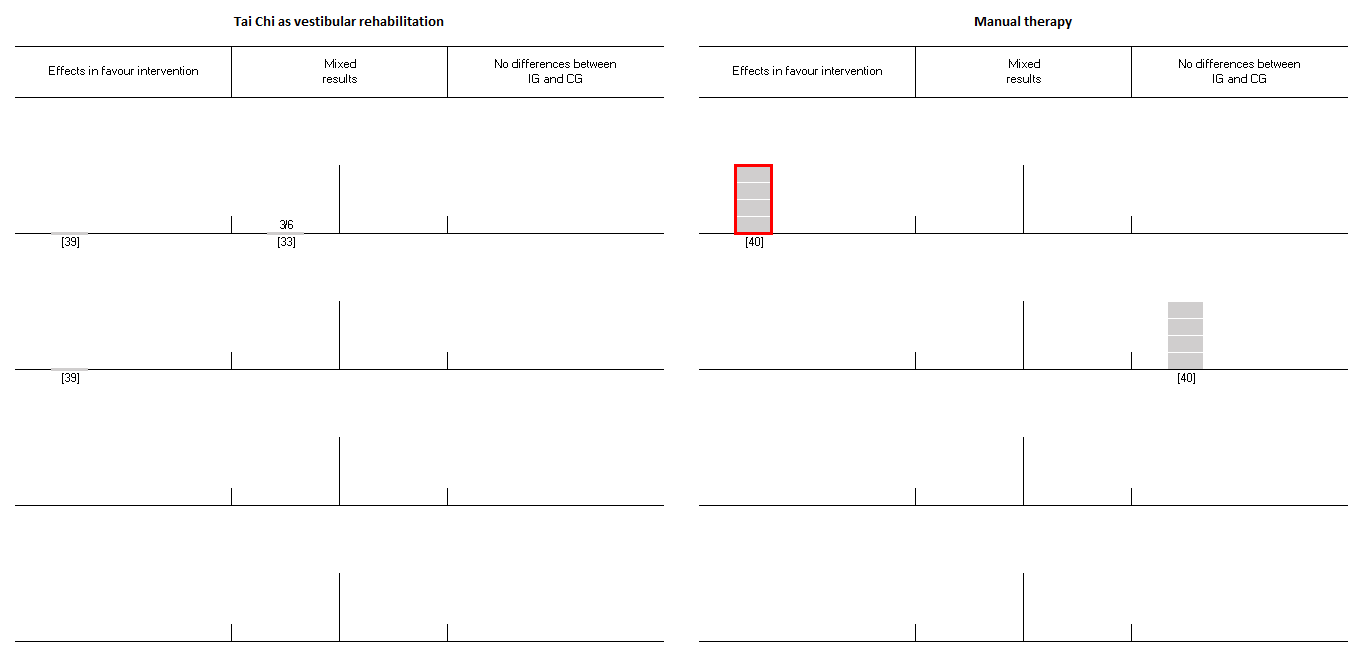


Methodologic quality of the study design is indicated by the height of the bar (the higher the bar the lower the risk of bias). The numbers below the bars indicate the reference number. The colour of the bar indicates the type of comparison (light grey – intervention compared versus no/sham intervention; black – versus usual care). "Mixed results" mean studies reported both, effects in favour of the intervention and no effect. Numbers on top of the bars specifies how many of the reported outcomes are reported with effects (number of outcomes with effects / number of outcomes). Red fringe indicates primary outcome.
