# Supplementary material for: Physical therapy interventions for older people with vertigo, dizziness and balance disorders addressing mobility and participation: a systematic review
Source: BMC Geriatr. 2020 Nov 23;20:494. doi: 10.1186/s12877-020-01899-9 (PMC7684969; doi:10.1186/s12877-020-01899-9)
Supplement: Supplementary file 7 — Additional file 7. Evidence tables rating quality of evidence and summary of findings. [file 12877_2020_1899_MOESM7_ESM.docx]

**Additional file 7** Evidence tables rating quality of evidence and summary of findings

1. Canal repositioning manoeuvre

| Outcome | | No. of participants  (studies) | **Quality assessment** | | | | | Overall quality of evidence | **Summary of findings** |
| --- | --- | --- | --- | --- | --- | --- | --- | --- | --- |
|  |  |  | Risk of bias | Inconsistency | Indirectness | Imprecision | Publication bias |  | Effects |
| Canal repositioning manoeuvre (Epley) alone compared to wearing a neck collar for 48 hours after manoeuvre | | | | | | | | | |
| Activities and participation | Impact of VDB on ADL (DHI) | 53  (1 RCT [31]) | Serious^a^ | Not serious | Not serious | Serious^b^ | Likely^a^ | $\oplus$OOO  Very low | ↔  No difference [31]. |
| Canal repositioning manoeuvre (Epley) alone compared to using a mini-vibrator placed on the mastoid of affected side during manoeuvre | | | | | | | | | |
| Activities and participation | Impact of VDB on ADL  (DHI) | 53  (1 RCT [31]) | Serious^a^ | Not serious | Not serious | Serious^b^ | Likely^a^ | $\oplus$OOO  Very low | ↔  No difference [31]. |
| ^a^Insufficient information  ^b^Narrative analysis was conducted, estimates are not precise  [ ] = Reference number  ADL = activities of daily living; DHI = Dizziness Handicap Inventory; RCT = Randomized controlled trial; VDB = vertigo, dizziness and balance disorders | | | | | | | | | |

1. Vestibular rehabilitation

| Outcome | | No. of participants  (studies) | **Quality assessment** | | | | | | Overall quality of evidence | **Summary of findings** |
| --- | --- | --- | --- | --- | --- | --- | --- | --- | --- | --- |
|  |  |  | Risk of bias | Inconsistency | Indirectness | Imprecision | Publication bias |  | | Effects |
| Vestibular rehabilitation compared to usual care | | | | | | | | | | |
| Body structure and function | **VDB Symptoms (VSS-SF**, dizziness frequency calendar) | 296  1 RCT [**35**, 49]) | Not serious | Not serious | Not serious | Serious^e^ | None | $\oplus\oplus\oplus$O  Moderate | | **↑** ↔  Advantage in VSS-SF [35]. No difference in dizziness frequency [49]. |
|  | Presence of psychological disorders (HADS, GAD7, PHQ-PC, PHQ-9) | 464  (2 RCT [35, 49]) | Not serious^a,c^ | Not serious | Not serious | Serious^e^ | None | $\oplus\oplus\oplus$O  Moderate | | ↔ ↔  No differences [35, 49]. |
|  | **Postural control** (CTSIB, **mCTSIB,** SOT, **LOS, RWS**) | 255  (3 RCTs [30, **44**, 47]) | Not serious^a,c^ | Not serious | Not serious^d^ | Serious^e^ | None^f^ | $\oplus\oplus\oplus$O  Moderate | | ↑ ↑ **↔ ↔** **↔** ↔ ↔ ↔  2 studies [30, 44] showed no difference in sensory integration in balance, 1 study [44] no difference in limits of stability and weight shift, 1 study [47] observed a better use of vestibular systems and no difference of visual and somatosensory systems. |
|  | Lower extremity strength and transfer sit to stand (STS, 5xSTS, strength) | 165  (1 RCT [44]) | Not serious^a^ | Not serious | Not serious | Serious^e^ | Very likely | $\oplus$OOO  Very low | | ↔  No difference [44]. |
| Activities and participation | Balance  (BBS, DGI, FRT, ABC) | 315  (3 RCT [30, 44, 47]; 1 nRCT [50]) | Not serious^a,b,c^ | Not serious | Not serious^d^ | Serious^e^ | None^f^ | $\oplus\oplus\oplus$O  Moderate | | ↑ ↑ ↑ ↑ ↑ ↑ ↔ ↔  2 studies [30, 47] observed an advantage and 1 study [50] no difference in static balance. 2 studies [30, 50] showed an advantage in dynamic balance. 1 study [44] observed an advantage and 1 study [47] no difference in functional balance. 1 study [30] showed an advantage in balance confidence. |
|  | **Impact of VDB on ADL**  (DHI) | 464  (2 RCT [35, **49**]) | Not serious^a,c^ | Not serious | Not serious | Serious^e^ | None | $\oplus\oplus\oplus$O  Moderate | | ↑ **↔**  1 study [35] showed an advantage and 1 study [49] showed no difference. |
|  | Mobility  (TUG, WA) | 255  (3 RCTs [30, 44, 47]) | Not serious^a,c^ | Not serious | Not serious^d^ | Serious^e^ | None^f^ | $\oplus\oplus\oplus$O  Moderate | | ↑ ↑ ↔  2 studies [44, 47] found an advantage,1 study [30] no difference, |
|  | Parkinson specific ADL  (UPDRS) | 225  (2 RCTs [30, 47]) | Serious^a,c^ | Not serious | Not serious | Serious^e^ | None | $\oplus\oplus$OO  Low | | ↔ ↔  2 studies [30, 47] found no difference. |
| Quality of life | (PDQ-39, AQoL, QoL) | 393  (3 RCTs [30, 44, 49] | Not serious^a,c^ | Not serious | Not serious^d^ | Serious^e^ | None^f^ | $\oplus\oplus\oplus$O  Moderate | | ↔ ↔  2 studies [30, 44] showed no difference |
| Falls | Fear of falling (VAS-FOF, MFES) | 225  (1 RCT [44],  1 nRCT [50]) | Serious^a,b,c^ | Not serious | Not serious | Serious^e^ | None^f^ | $\oplus\oplus$OO  Low | | ↔ ↑  1 study [44] found no difference.1 study [50] an advantage. |
|  | Frequency of falls | 60  (1 RCT [49], 1 nRCT [50]) | Serious^a,b,c^ | Not serious | Not serious | Serious^e^ | None | $\oplus\oplus$OO  Low | | ↔ ↔  No difference [49, 50]. |
| Vestibular rehabilitation compared to no intervention | | | | | | | | | | |
| Body structure and functions | **Postural control** (**SOLEO, SOLEC**, tandem standing with eyes open and closed, SOT, LOS) | 282  (2 RCT [**37**, 51], 1 nRCT [36]) | Serious^a,b^ | Not serious | Not serious^d^ | Serious^e^ | Likely^f^ | $\oplus$OOO  Very low | | ↑ **↔ ↔** ↔ ↔ ↔ ↔ ↔ ↔ ↔ ↔ ↔ ↔ ↔ ↔  1 study [36] found an advantage in SOLEC, another study [37] not. 2 studies [36, 37] observed no difference in SOLEO, tandem standing with eyes open and closed and 1 study [37] in postural sway and vibration sense. 1 study [51] reported no difference in sensory integration and limits of stability. |
|  | Nystagmus  (Head shake test) | 85  (1 RCT [37]) | Not serious^a^ | Not serious | Not serious | Serious^e^ | Likely | $\oplus\oplus$OO  Low | | ↔  No difference [37]. |
|  | Lower extremity strength and transfer sit to stand (5xSTS) | 85  (1 RCT [37]) | Not serious^a^ | Not serious | Not serious | Serious^e^ | Likely | $\oplus\oplus$OO  Low | | ↔  No difference [37]. |
|  | Self-related health (EQ5D-VAS) | 85  (1 RCT [37]) | Not serious^a^ | Not serious | Not serious | Serious^e^ | Likely | $\oplus\oplus$OO  Low | | ↔  No difference [37]. |
| Activities and participation | Impact of VDB on ADL  (DHI) | 197  (1 RCT [51], 1 nRCT [36]) | Serious^a,b^ | Not serious | Not serious | Serious^e^ | Likely^f^ | $\oplus$OOO  Very low | | ↔ ↔  2 studies [36, 51] observed no difference. |
|  | **Gait** (**walking variations**, TUG) | 282  (1 RCT [**37**], 1 nRCT [36]) | Serious^a,b^ | Not serious | Not serious^d^ | Serious^e^ | Likely^f^ | $\oplus$OOO  Very low | | ↑ **↔** ↔ ↔ ↔ ↔  1 study [36] observed an advantage in walking heel to toe on a line, 1 study [37] not. 2 studies [36, 37] found no difference in steps outside when walking a figure of eight. 1 study [36] observed no difference in walking as fast as possible for 30 m with one turn after 15 m. No difference in TUG [51]. |
| Falls | Frequency of falls (risk of falls, FES-I) | 197  (1 RCT [51], 1 nRCT [36]) | Serious^a,b^ | Not serious | Not serious | Serious^e^ | Likely^f^ | $\oplus$OOO  Very low | | ↔ ↔  No difference [36, 51]. |
| Otago exercise programme in groups compared to the Otago exercise programme at home | | | | | | | | | | |
| Body structure and functions | Lower extremity strength and transfer sit to stand (5xSTS) | 125  (1 RCT [48]) | Not serious^a^ | Not serious | Not serious | Serious^e^ | Very likely | $\oplus$OOO  Very low | | ↑  Advantage [48]. |
| Activities and participation | **Balance**  **(BBS)** | 125  (1 RCT [**48**]) | Not serious^a^ | Not serious | Not serious | Serious^e^ | Very likely | $\oplus$OOO  Very low | | **↔**  No difference [48]. |
|  | Mobility  (TUG) | 125  (1 RCT [48]) | Not serious^a^ | Not serious | Not serious | Serious^e^ | Very likely | $\oplus$OOO  Very low | | ↑  Advantage [48]. |
| Quality of life | (SF-36) | 125  (1 RCT [48]) | Not serious^a^ | Not serious | Not serious | Serious^e^ | Very likely | $\oplus$OOO  Very low | | ↔  No difference [48]. |

| Falls | (FES-I) | 125  (1 RCT [48]) | Not serious^a^ | Not serious | Not serious | Serious^e^ | Very likely | $\oplus$OOO  Very low | ↔  No difference [48]. |
| --- | --- | --- | --- | --- | --- | --- | --- | --- | --- |
| VR in addition to CRM compared to CRM alone | | | | | | | | | |
| Body structure and functions | **Postural control** (**mCTSIB**, US sway, **LOS**, tandem) | 16  (1 RCT [**41**]) | Not serious^a^ | Not serious | Not serious | Serious^e^ | None | $\oplus\oplus\oplus$O  Moderate | **↔ ↔** ↔ ↔  No difference in sensory integration and limits of stability (movement velocity), advantage in maximum excursion and in tandem end sway [41]. |
|  | Intensity of dizziness (VAS) | 16  (1 RCT [41]) | Not serious^a^ | Not serious | Not serious | Serious^e^ | None | $\oplus\oplus\oplus$O  Moderate | ↔  No difference [41]. |
| Activities and participation | **Balance**  (**DGI**) | 16  (1 RCT [**41**]) | Not serious^a^ | Not serious | Not serious | Serious^e^ | None | $\oplus\oplus\oplus$O  Moderate | **↑**  Advantage [41]. |
|  | Mobility (WA speed) | 16  (1 RCT [41]) | Not serious^a^ | Not serious | Not serious | Serious^e^ | None | $\oplus\oplus\oplus$O  Moderate | ↔  No difference [41]. |
|  | Impact of VDB on ADL  (DHI) | 16  (1 RCT [41]) | Not serious^a^ | Not serious | Not serious | Serious^e^ | None | $\oplus\oplus\oplus$O  Moderate | ↔  No difference [41]. |
| Multimodal version versus the conventional version of the Cawthorne-Cooksey programme | | | | | | | | | |
| Body structure and functions | Strength (STS, handgrip strength) | 82  1 RCT [42] | Not serious^a^ | Not serious | Not serious | Serious^e^ | None | $\oplus\oplus\oplus$O  Moderate | ↔  No difference [42]. |
| Activities and participation | **Balance** **and mobility** (**DGI**, TUG, FRT, tandem stand) | 82  1 RCT [**42**] | Not serious^a^ | Not serious | Not serious | Serious^e^ | None | $\oplus\oplus\oplus$O  Moderate | **↔**  No difference [42]. |
| Falls | Likelihood of falls (Romberg, fall rate) | 82  1 RCT [42] | Not serious^a^ | Not serious | Not serious | Serious^e^ | None | $\oplus\oplus\oplus$O  Moderate | ↔  No difference [42]. |
| VR with computer dynamic posturography exercises to exposure to optokinetic stimuli and exercises at home based on the Cawthorne-Cooksey programme | | | | | | | | | |
| Body structure and functions | Postural control (SOT) | 139  (1 RCT [51]) | Not serious^a^ | Not serious | Not serious | Serious^e^ | None | $\oplus\oplus\oplus$O  Moderate | Information missing |
| Activities and participation | Impact of VDB on ADL  (DHI) | 139  (1 RCT [51]) | Not serious^a^ | Not serious | Not serious | Serious^e^ | None | $\oplus\oplus\oplus$O  Moderate | Information missing |
|  | Mobility (TUG) | 139  (1 RCT [51]) | Not serious^a^ | Not serious | Not serious | Serious^e^ | None | $\oplus\oplus\oplus$O  Moderate | Information missing |
| Falls | (FES-I) | 139  (1 RCT [51]) | Not serious^a^ | Not serious | Not serious | Serious^e^ | None | $\oplus\oplus\oplus$O  Moderate | Information missing |
| ^a^Insufficient information  ^b^Contains study without randomization [50, 36]  ^c^Attrition unbalanced between groups and patients aware of assignment  ^d^Population of two studies explicitly suffered from Parkinson Disease [30, 47] or from fall-related wrist fractures [37] but authors estimate comparability  ^e^Narrative analysis was conducted, estimates are not precise  ^f^Contains study with very likely publication bias [44, 36]  **Bold and underlined letters** = primary outcome in one study  ADL = activities of daily living; RCT = Randomized controlled trial; VDB = vertigo, dizziness and balance disorders | | | | | | | | | |

1. Computer assisted vestibular rehabilitation

| Outcome | | No. of participants  (studies) | **Quality assessment** | | | | | Overall quality of evidence | **Summary of findings** |  |
| --- | --- | --- | --- | --- | --- | --- | --- | --- | --- | --- |
|  |  |  | Risk of bias | Inconsistency | Indirectness | Imprecision | Publication bias |  | Effects |  |
| Computer assisted vestibular rehabilitation compared to usual care (Wii Fit programme to conventional physical therapy in comparison to conventional physiotherapy) | | | | | | | | | |  |
| Body structure and functions | Postural control (baropodometry, stabilometry) | 20  (1 RCT [46]) | Not serious^a^ | Not serious | Not serious | Serious^b^ | Very likely | $\oplus$OOO  Very low | ↔  No differences [46]. | |
| Activities and participation | Balance, Mobility and independence | 20  (1 RCT [46]) | Not serious^a^ | Not serious | Not serious | Serious^b^ | Very likely | $\oplus$OOO  Very low | ↔  No differences [46]. | |
| Computer assisted vestibular rehabilitation compared to no intervention | | | | | | | | | | |
| Body structure and functions | Postural control (SOT) | 42  (1 RCT [45]) | Not serious^a^ | Not serious | Not serious | Serious^b^ | Very likely | $\oplus$OOO  Very low | ↔  No differences [45]. | |
| Activities and participation | Dual tasking (VRT) | 42  (1 RCT [45]) | Not serious^a^ | Not serious | Not serious | Serious^b^ | Very likely | $\oplus$OOO  Very low | ↔  No differences [45]. | |
| Virtual reality-based Wii Fit training with subsequent treadmill training compared to fall-prevention education with no structured programme | | | | | | | | | | |
| Body structure and functions | Gait parameters (velocity, stride length) | 36  (1 RCT [38]) | Not serious^a^ | Not serious | Not serious | Serious^b^ | None | $\oplus\oplus\oplus$O  Moderate | ↑  Advantages [38]. | |
|  | Lower extremity strength | 36  (1 RCT [38]) | Not serious^a^ | Not serious | Not serious | Serious^b^ | None | $\oplus\oplus\oplus$O  Moderate | ↑ ↑ ↑ ↑ ↑ ↔  Advantages in hipflexors, knee flexors and extendors, ankle dorsiflexors and plantarflexors. No difference in hip extensors [38]. | |
|  | Postural control (SOT) | 36  (1 RCT [38]) | Not serious^a^ | Not serious | Not serious | Serious^b^ | None | $\oplus\oplus\oplus$O  Moderate | ↑ ↑ ↔  Advantage in visual and vestibular ratios. No difference in somatosensory ratios [38]. | |
| Activities and participation | Balance (FGA) | 36  (1 RCT [38]) | Not serious^a^ | Not serious | Not serious | Serious^b^ | None | $\oplus\oplus\oplus$O  Moderate | ↑  Advantage [38]. | |
| Home exercises supported by the “Move it to improve it” (Mitii) computer programme compared to a printed home programme | | | | | | | | | | |
| Body structure and functions | VDB symptoms (Intensity VAS, motion-promoted dizziness) | 63  (1 RCT [43]) | Not serious^a^ | Not serious | Not serious | Serious^b^ | None | $\oplus\oplus\oplus$O  Moderate | ↔  No difference [43]. | |
|  | Lower extremity strength (Chair stand test) | 63  (1 RCT [43]) | Not serious^a^ | Not serious | Not serious | Serious^b^ | None | $\oplus\oplus\oplus$O  Moderate | ↔  No difference [43]. | |
| Activities and participation | **Balance** (**One-leg-stand-test,** DGI) | 63  (1 RCT [**43**]) | Not serious^a^ | Not serious | Not serious | Serious^b^ | None | $\oplus\oplus\oplus$O  Moderate | **↔** ↔  No difference in static and dynamic balance [43]. | |
|  | Impact of VDB on ADL (DHI) | 63  (1 RCT [43]) | Not serious^a^ | Not serious | Not serious | Serious^b^ | None | $\oplus\oplus\oplus$O  Moderate | ↔  No difference [43]. | |
| Quality of life | (SF-12) | 63  (1 RCT [43]) | Not serious^a^ | Not serious | Not serious | Serious^b^ | None | $\oplus\oplus\oplus$O  Moderate | ↔  No difference [43]. | |
| Remotely supervised in-home virtual reality balance training (TeleWii) compared to in-clinic sensory integration balance training | | | | | | | | | | |
| Activities and participation | **Balance** and Mobility (**BBS**, ABC, 10-MW, DGI) | 76  (1 RCT [**34**]) | Not serious^a^ | Not serious | Not serious | Serious^b^ | None | $\oplus\oplus\oplus$O  Moderate | **↔** ↔ ↔ ↔  No difference [34]. | |
| Quality of life | PDQ-39 | 76  (1 RCT [34]) | Not serious^a^ | Not serious | Not serious | Serious^b^ | None | $\oplus\oplus\oplus$O  Moderate | ↔  No difference [34]. | |
| Falls | Falls | 76  (1 RCT [34]) | Not serious^a^ | Not serious | Not serious | Serious^b^ | None | $\oplus\oplus\oplus$O  Moderate | ↔  No difference [34]. | |
| ^a^Insufficient information  ^b^Narrative analysis was conducted, estimates are not precise  ADL = activities of daily living; RCT = Randomized controlled trial; VDB = vertigo, dizziness and balance disorders | | | | | | | | | | |

1. Tai Chi as vestibular rehabilitation

| Outcome | | No. of participants  (studies) | **Quality assessment** | | | | | Overall quality of evidence | **Summary of findings** |  |
| --- | --- | --- | --- | --- | --- | --- | --- | --- | --- | --- |
|  |  |  | Risk of bias | Inconsistency | Indirectness | Imprecision | Publication bias |  | Effects |  |
| TCVR compared to no/sham intervention | | | | | | | | | |  |
| Body structure and functions | Postural control (passive knee joint repositioning test, SOT, LOS) | 80  (2 RCTs [33, 39]) | Serious^a^ | Not serious | Not serious | Serious^e^ | None^f^ | $\oplus\oplus$OO  Low | ↑ ↑ ↑ ↑ ↑ ↑ ↔ ↔ ↔  1 study [33] showed an advantage in repositioning test, advantage in visual ratio and vestibular ratio of SOT and no difference in somatosensory ratio. 1 study [39] observed advantages in forward, backward and in maximal sway area, but not right- and leftwards. | |
|  | Lower extremity strength (concentric isokinetic) | 40  (1 RCT [33]) | Not serious^a^ | Not serious | Not serious | Serious^e^ | None | $\oplus\oplus\oplus$O  Moderate | ↔ ↔  No difference in knee extensor or flexor strength [33]. | |
| Activities and participation | Mobility (8 foot up and go test) | 40  (1 RCT [39]) | Serious^a^ | Not serious | Not serious | Serious^e^ | Likely | $\oplus$OOO  Very low | ↑  Advantage [39]. | |
| TCVR compared to breathing and stretching exercises | | | | | | | | | | |
| Body structure and functions | **Postural control (LOS, SOT)** | 136  (1 RCT [**32**]) | Not serious^a^ | Not serious | Not serious | Serious^e^ | Very likely | $\oplus$OOO  Very low | **↑ ↑ ↑ ↑ ↑ ↔ ↔ ↔ ↔ ↔ ↔ ↔ ↔ ↔ ↔ ↔**  Advantage in reaction time of nonaffected side in LOS.  No difference in reaction time of all other sides in LOS.  Advantage in end-point excursion of non-affected and affected side and backwards and forwards in LOS.  No difference in all conditions of equilibrium score and sensory ratios of SOT. | |
| Activities and participation | Mobility (TUG) | 136  (1 RCT [32]) | Not serious^a^ | Not serious | Not serious | Serious^e^ | Very likely | $\oplus$OOO  Very low | ↔  No difference [32]. | |

1. Manual therapy

| Outcome | | No. of participants  (studies) | **Quality assessment** | | | | | Overall quality of evidence | **Summary of findings** |  |
| --- | --- | --- | --- | --- | --- | --- | --- | --- | --- | --- |
|  |  |  | Risk of bias | Inconsistency | Indirectness | Imprecision | Publication bias |  | Effects |  |
| Manual therapy (SNAGs) versus sham intervention | | | | | | | | | |  |
| Body structure and functions | **Dizziness** and neck pain (**intensities,** frequency) | 86  (1 RCT [**40**]) | Not serious^a^ | Not serious | Not serious | Serious^e^ | Very likely | $\oplus$OOO  Very low | **↑** ↑ ↔  Advantage in **VAS dizziness** and dizziness frequency.  No difference in VAS pain. | |
| Activities and participation | Impact of VDB an ADL (DHI) | 86  (1 RCT [40]) | Not serious^a^ | Not serious | Not serious | Serious^e^ | Very likely | $\oplus$OOO  Very low | No differences [40]. | |
| Different forms of Manual therapy: SNAGs compared to Maitland mobilizations | | | | | | | | | | |
| Body structure and functions | **Dizziness** and neck pain (**intensities,** frequency) | 86  (1 RCT [**40**]) | Not serious^a^ | Not serious | Not serious | Serious^e^ | Very likely | $\oplus$OOO  Very low | **↔** ↔ ↔  No differences [40]. | |
| Activities and participation | Impact of VDB an ADL (DHI) | 86  (1 RCT [40]) | Not serious^a^ | Not serious | Not serious | Serious^e^ | Very likely | $\oplus$OOO  Very low | ↓  Disadvantage [40]. | |
